# COVID-19 epidemiology and changes in health service utilization in Uganda’s refugee settlements during the first year of the pandemic

**DOI:** 10.1186/s12889-022-14305-3

**Published:** 2022-10-17

**Authors:** Chiara Altare, Natalya Kostandova, Jennifer OKeeffe, Emmanuel Omwony, Ronald Nyakoojo, Julius Kasozi, Paul B. Spiegel

**Affiliations:** 1 Johns Hopkins Bloomberg School of Public Health, 615 N Wolfe str, Baltimore, MD 21205 USA; 2 Johns Hopkins Center for Humanitarian Health, 615 N Wolfe str, Baltimore, MD 21205 USA; 3 United Nations High Commissioner for Refugees, Kampala, Uganda

**Keywords:** Health service utilization, COVID-19 pandemic, Refugee settlements, Uganda

## Abstract

**Background:**

The COVID-19 pandemic has been characterized by multiple waves with varying rates of transmission affecting countries at different times and magnitudes. Forced displacement settings were considered particularly at risk due to pre-existing vulnerabilities. Yet, the effects of COVID-19 in refugee settings are not well understood. In this study, we report on the epidemiology of COVID-19 cases in Uganda’s refugee settlement regions of West Nile, Center and South, and evaluate how health service utilization changed during the first year of the pandemic.

**Methods:**

We calculate descriptive statistics, testing rates, and incidence rates of COVID-19 cases in UNHCR’s line list and adjusted odds ratios for selected outcomes. We evaluate the changes in health services using monthly routine data from UNHCR’s health information system (January 2017 to March 2021) and apply interrupted time series analysis with a generalized additive model and negative binomial distribution, accounting for long-term trends and seasonality, reporting results as incidence rate ratios.

**Findings:**

The first COVID-19 case was registered in Uganda on March 20, 2020, and among refugees two months later on May 22, 2020 in Adjumani settlement. Incidence rates were higher at national level for the general population compared to refugees by region and overall. Testing capacity in the settlements was lower compared to the national level. Characteristics of COVID-19 cases among refugees in Uganda seem to align with the global epidemiology of COVID-19. Only hospitalization rate was higher than globally reported. The indirect effects of COVID-19 on routine health services and outcomes appear quite consistent across regions. Maternal and child routine and preventative health services seem to have been less affected by COVID-19 than consultations for acute conditions. All regions reported a decrease in consultations for respiratory tract infections.

**Interpretation:**

COVID-19 transmission seemed lower in settlement regions than the national average, but so was testing capacity. Disruptions to health services were limited, and mainly affected consultations for acute conditions. This study, focusing on the first year of the pandemic, warrants follow-up research to investigate how susceptibility evolved over time, and how and whether health services could be maintained.

**Supplementary Information:**

The online version contains supplementary material available at 10.1186/s12889-022-14305-3.

## Background

The global pandemic caused by the SARS-CoV-2 virus has been characterized by multiple waves with varying rates of transmission affecting countries at different times and magnitudes. Humanitarian and forced displacement settings were considered particularly at risk due to pre-existing vulnerabilities such as fragile living conditions, limited access to water and sanitation facilities, high population density, and limited available space, and dependence upon external funding [1, 2]. These factors cast doubts about governments' and the international community’s readiness and response capacity to protect affected populations in forced displacement settings from COVID-19, as ensuring access to sufficient testing, infection prevention and control measures for refugees requires a comprehensive approach to ensure the safety of citizens and non-citizens [3]. Furthermore, previous large-scale epidemics (e.g., Ebola in West Africa and Cholera in Yemen) showed increased vulnerabilities and negative outcomes from other communicable and non-communicable diseases as attention and funding were diverted toward the outbreaks [4]. Maintaining essential health services became a priority.

The number of cases in such settings has increased over time [5], yet reported cases and deaths have not reached high levels of other countries like Brazil or India [5], nor the gloomy scenarios from initial modeling exercises [6, 7]. A recent meta-analysis of seroprevalence studies conducted in African countries reported higher seroprevalence than indicated by surveillance data, which, while not surprising given the limited testing capacity, points to higher exposure and acquired protection from the virus [8]. Yet, despite increasing evidence about COVID-19 and its spread globally, few studies about the direct and indirect effects of the virus in humanitarian and forced displacement settings exist. Articles about refugee populations published so far include modeling studies estimating the number of cases and the role of non-pharmaceutical measures to control the spread of the disease [1, 6, 9, 10]; reviews of context-specific vulnerabilities and possible impacts in conjunction with appeals for action and integration of refugees in national health responses [11, 12, 13]; and assessments of knowledge and attitude regarding COVID-19 [14, 15]. One qualitative study reported on the impact of COVID-19 on utilization and access to maternal services among eleven refugee women in urban Kenya [16]. Another article estimated the mortality rate and seroprevalence of SARS-CoV-2 antibodies among residents in a refugee camp in Kenya as well as compared consultations at health centers before and during the pandemic [17].

Uganda hosts the fourth highest number of refugees worldwide, after Turkey, Colombia and Pakistan, and the highest in Africa [18]. Following a significant reduction in 2018, new influxes of refugees have steadily increased since October 2018, reaching more than 1.4 million as of March 31, 2021 [19]. The first case of COVID-19 in Uganda was recorded on March 20, 2020. A few days earlier, the Government of Uganda had instituted public health measures to prevent infections, such as restrictions and ban on public gatherings and movements, suspension of public transport, closure of schools and other public spaces, night curfew and requirements to physically distance and wear masks. A temporary ban on the entry and exit of foreign nationals (including refugees and asylum seekers) was announced on March 22, 2020. The majority of the restrictions were progressively eased from beginning of June 2020 [20]. While all these measures applied to the refugee settlements too [21] (details in supplementary material), some were re-instated when the situation worsened in specific settlements. For example, Kyangwali settlement was put under lockdown at the end of August 2020 after an increase in infections among both humanitarian aid workers and refugees [22]. Other measures were likely only implemented in settlements, such as soap and hand sanitizer distribution, or setting up of handwashing stations [23]. PCR testing capacity was initially available only in Kampala and was then decentralized to two locations in Adjumani (West Nile) and Mutukula (near Nakivale settlement) which served as testing centers in these regions between March and June 2020. PCR testing capacity was established in the country's various regions as of June 2020.

In this study, we report on the epidemiology of COVID-19 cases in Uganda’s refugee settlements and evaluate the effects of COVID-19 and its related response measures on routine health services during the first year of the pandemic.

## Methods

### Study setting

The majority of the 1.4 million refugees in Uganda live in 12 main settlements across the country, where refugees live alongside local communities: about 60% of the refugees are in Northern Uganda or West Nile, 25% in southwestern/ southern Uganda, 13% in central Uganda, and 6% in Kampala (Fig S1 Supplementary material) [24]. The majority (60%) of the refugee population is under the age of 18 years, and only 3% are 60 years old or more. All settlements were included in the analysis, while refugees living in Kampala were excluded. The leading causes of illness and death among the refugees are malaria, respiratory and diarrheal diseases. Mortality rates have, however, remained low since 2014 [25]. The refugee health and nutrition response is guided by the Uganda National Integrated Health Response Plan for Refugees & Host Communities [25] and the United Nations High Commissioner for Refugees (UNHCR) Public Health Strategic Plan 2018–2022 [26]. Health services are provided by the national authorities with support of both humanitarian and development actors and are accessible by both nationals and refugees. Health service delivery in the settlements focuses on disease prevention and community initiatives; sexual, reproductive, maternal, neonatal child and adolescent health; and prevention, management and control of communicable and non-communicable diseases. Primary health care facilities (levels II to IV) are located in the settlements, with most of the population residing within a 5 km radius to ensure all communities are served. Outreach activities are conducted by health facility personnel to reach remote areas, and community health workers are in charge of referrals and surveillance activities. Communities in the settlements rely on regional and national level referral hospitals (usually outside of the settlements) for tertiary level procedures. Ambulance services are provided in all settlements to transport patients to and from referral health facilities. Table 1 provides key information about the settlements.

**Table 1:** Refugee settlements in Uganda regrouped by region

Settlement	Mid point population size (March 2020 –March 2021)	Country of origin^a^	Opening date	Number of health facilities by level			
				**Total**	**Level II**	**Level III**	**Level IV**
**West Nile region**							
Adjumani^b^	203,517	SSD	2014	17	12	4	1
BidiBidi	233,892	SSD	Aug 2016	17	1	16	
Imvepi	66,633	SSD	Feb 2017	6	5	1	
Rhino camp	119,873	SSD	1980	12	5	5	2
Palabek	54,738	SSD	2016	3	2	1	
Palorinya	123,034	SSD	Dec 2016	9	4	5	
**Center region**							
Kiryandongo	67,892	SSD	1990 and reopened in 2014	3	2	2	
Kyangwali	119,872	DRC	1960	10	5	4	1
**South region**							
Kyaka II	124,233	DRC	2005	3		3	
Oruchinga	7,936	DRC, BDI, RWA	1959	2	1	1	
Nakivale	124,676	DRC, BDI, SOM	1958	6	4	1	1
Rwamwanja	72,562	DRC	2012	6	5	1	
**Other (excluded from analysis)**							
Kampala^c^	91,193	SOM, DRC, others	–				

Sources of data: i) UNHCR population data

^a^ SSD: South Sudan; DRC: Democratic Republic of the Congo; BDI: Burundi; RWA: Rwanda; SOM: Somalia

^b^ Adjumani settlements encompasses 18 smaller settlements

^c^ Kampala is not a settlement; rather this represents the estimated number of refugees living in the Uganda capital Kampala. Kampala was not included in the study

### Data sources and study outcomes

The study used two primary sets of data by UNHCR: i) the COVID-19 line list, and ii) routine health data from UNHCR’s health information system (HIS). Data were initially recorded at settlement level and subsequently aggregated by region (West Nile, South and Center). This regional approach was chosen for several reasons: i) it allowed for mobility between settlements in the pre-COVID-19 period to be taken into account. Under Uganda’s refugee policy [27], refugees can move freely, have access to land in the settlement where they are registered, and can access services in other settlements. Especially in West Nile region where numerous proximate settlements have been created to host refugees in the same area, mobility cannot be excluded nor can it be tracked. Furthermore, not all camps opened at the same time and new camps’ health facilities were not operational right away; consequently, refugees used services offered in the other settlements. Finally, it is not uncommon for refugees to be registered in one camp and reside in another settlement or use another settlements’ services. Consequently, analyzing health care utilization at the regional level better captures population dynamics and utilization within the region; ii) it allows for more stable pre-COVID-19 trends, and therefore, for a better counterfactual in the interrupted times series analysis; and iii) it aligns with UNHCR’s operations, which facilitates the use of the findings for the agency’s programmatic and operational purposes.

A COVID-19 line list (i.e., a table that contains key information about each case in an outbreak) was established by UNHCR in each settlement and included laboratory-confirmed COVID-19 cases between March 1, 2020, and March 31, 2021. While the variables included in the line lists varied by settlement (Table S3), the line list included some combination of patient demographics, SARS-CoV-2 test data, presence of comorbidities, isolation and hospitalization, exposure risks due to occupation, disease outcome, and a number of contacts followed by case. Only one settlement collected information on the epidemiological link of the cases (i.e., the possible source of infection). The number of cases from the line list was used to calculate monthly and overall incidence rates for refugees in settlements. In addition to the line list, aggregated number of conducted tests were obtained to calculate the overall testing rate and percent positive by settlement for the entire period. The number of tests conducted per month by the settlement is unavailable for the first wave (November 2020 to January 2021). National level (Uganda) data on COVID-19 cases for the study period and population estimates were obtained from the Johns Hopkins COVID-19 Resource Center [28]; national level testing data were obtained from Our World in Data [29]. Aggregated number of tests and confirmed COVID-19 cases in the districts where settlements are located were obtained from the district health offices; however, monthly data were unavailable.

UNHCR’s HIS in Uganda includes monthly data from health facilities by settlement, which we added up to generate a monthly value for each settlement region. For this study, we extracted the following variables: number of outpatient consultations; first antenatal care (ANC1) visits; deliveries attended by skilled health workers; contraceptive prevalence; Diphtheria, Pertussis, Tetanus (DPT1) vaccination coverage; coverage of full vaccination; respiratory tract infections (RTIs; disaggregated by type: upper respiratory tract infection (URTI), and lower respiratory tract infection (LRTI) and influenza-like illnesses (ILI)); consultations for diarrheal diseases; consultations for malaria; and mortality. Complete definitions of indicators are provided in the Supplementary material (Table S1). The study covers the period from January 1, 2017, to March 31, 2021.

### Statistical analysis

Descriptive statistics were calculated to describe COVID-19 case epidemiology. Comparisons of categorical variables were made with chi-square tests or Fisher’s exact tests; comparisons of continuous variables used t-tests to detect differences in means between two categories (sex), and analysis of variance (ANOVA) tests to detect differences in means between multiple categories (age groups). Odds ratios for selected outcomes were calculated using generalized linear models (with the binomial family link) and controlling for covariates: sex, age, and displacement status. *P*-values less than 0.05 were considered statistically significant. Comparisons between regions and the host countries were explored. Analysis was conducted in R (Version 4.1.0) using RStudio v1.4.1106 [30].

We used Interrupted Time Series to evaluate changes in rates of consultations and other outcomes during the COVID-19 period. The generalized additive model with first-order autoregression was fit for each region as follows:

$${y}_{i}=NB({y}_{i}|{\mu }_{i}, \theta )$$

$${log(\mu}_i)=\alpha_i+offset(log\left({population}_i\right))+\beta_1{COVID}_i+\beta_2{Month\;since\;COVID}_i+s({Centered\;Month}_i)+s(Month,bs=\text{cc},k=12)+\varepsilon_i$$

where $y$ is the outcome of interest, assumed to come from a negative binomial (NB) distribution with parameters ${\mu }_{i}$ and $\theta$; ${Population}_{i}$ is the number of people at risk or eligible to access relevant services at the time *i*; *COVID*_i_ is a binary variable (0 if month *i* is in the pre-COVID-19 period, and 1 if month *i* is in COVID-19 period); *Month since COVID*_i_ is time in months since the beginning of COVID-19 period (April 2020)*; s(Centered Month*_*i*_*)* is a smooth term, where *Centered Month*_*i*_ is the month number, centered at beginning of the COVID-19 period, which accounts for longer-term trend; and $s(Month, bs={\text{cc}},k=12)$ is a smooth term with cubic splines to capture 12-month seasonality cycle, where *Month* is a calendar month (from 1 to 12).

For services where seasonality was unlikely to be a factor, we used a model without seasonal dummy terms. For each indicator, we assessed possible lag using the autocorrelation function for up to 6 months; where a non-zero lag was observed, we ran the lagged model and presented those results in the main analysis; results comparing the model with and without lags are presented in Supplementary material for each indicator.

The model was fit using *mgcv* function in R [31]. We report parameter estimates using incidence rate ratios (IRR) and related 95% CI. ${\beta }_{1}$ estimates an immediate change in outcome at the beginning of the COVID-19 period (i.e., a change in level, or a step); ${\beta }_{2}$ estimates a change in slope in the evolution of outcome over time. Counterfactual values are predicted by setting values of ${COVID}_{i}$ and ${Month\ since\ COVID}_{i}$  to 0, and forecasting the model for 12 months of the COVID-19 period. Model diagnostics for each indicator are presented in Supplementary material.

## Results

### COVID-19 epidemiology

The first COVID-19 case reported in a settlement occurred on March 23, 2020, among nationals, and on May 22, 2020, among refugees. From the beginning of the outbreak until March 31, 2021, a total of 1,001 cases were reported from the settlements, of which 728 were among nationals, and 271 were among refugees. The two remaining cases were among non-refugee foreigners, and were excluded from the analysis. 

**Fig. 1:**
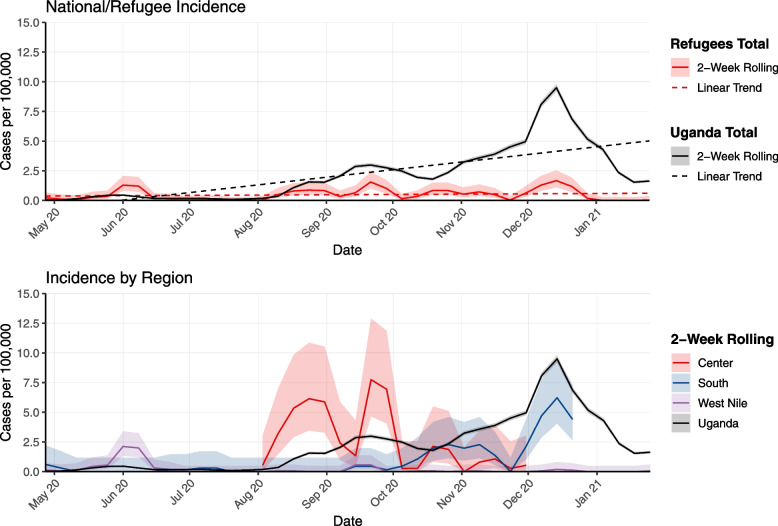
COVID-19 incidence rate over time in refugee settlements from May 2020 to March 2021 (2 week rolling average): aggregated incidence for refugees vs national level (upper panel); disaggregated by region vs national (lower panel)

Table 2 summarizes the proportion tested, percent positive, and incidence among refugees by region. The proportion tested was lower in the settlements in the regions than at the national level. West Nile settlements had the highest proportion tested (per 100,000) at 693.5 [95% CI 675.6 – 711.9] and the settlements in the South the lowest at 238.6 [95%CI 222.5 – 255.9] with the Uganda national proportion tested (per 100,000) at 1,042.7 [95%CI 1039.7 – 1045.6]. The percent positive among refugees from the South region was higher than the percent positive among Uganda nationals (13.5% [South] vs 8.1% [Uganda]), while the percent positive in the Center and West Nile were lower than among Uganda nationals (7.3% [Center] and 1.5% [West Nile] vs 8.1% [Uganda]). Incidence among refugees was lower than Uganda national level in all regions (47.0 [95%CI 38.2 – 57.9] [Center], 32.5 [95%CI 26.9 – 39.3] [South], and 9.5 [95%CI 7.6 – 11.9] [West Nile] vs 89.8 [95%CI 88.9 – 90.7] [Uganda]) (Fig. 1).

**Table 2:** Proportion tested (per 100,000), percent positive and incidence among refugees (by region) and nationals, Uganda, March 23, 2020—March 31, 2021

	**Refugees**				**Nationals**
	**Center**	**South**	**West Nile**	**Total**	**Total**
Population^a^	187,259	329,406	801,687	1,318,351	45,741,008
Confirmed COVID-19 cases^b^	94	106	83	283	41,077
Tests	1,284	786	5,560	7,630	476,930
Proportion Tested (per 100,000)^c^ [95% CI]	685.7 [649.3 – 724.1]	238.6 [222.5 – 255.9]	693.5 [675.6 – 711.9]	578.8 [566.0 – 591.9]	1,042.7 [1039.7 – 1045.6]
Percent Positive (%)^c^	7.3	13.5	1.5	3.7	8.1
Confirmed cases (from line list)^d^	88	107	76	271	41,077
Incidence rate (per 100,000) [95% CI]^d^	47.0 [38.2–57.9]	32.5 [26.9–39.3]	9.5 [7.6–11.9]	20.6 [18.3–23.2]	89.8 [88.9–90.7]

Sources of data: ^i^ UNHCR population data, ^ii^ Johns Hopkins Corona Virus Resource Center [28]; ^iii^ Number and percent positive tests: UNHCR testing data for refugee settlements/regions and OWiD for national level [29]; Population: UNHCR population data; ^iv^ Confirmed cases: UNHCR line list from refugee settlements for cases; Population: UNHCR population data

Table 3 summarizes descriptive results. The majority of the cases occur among men (56.8%, *n* = 567), in the Center region (53.6%, *n* = 535). The most affected age group was 18–29 years (34.6%, *n* = 345). The completeness of each variable varies and is shown in Table S3.

**Table 3:** Individual level characteristics of COVID-19 cases among nationals and refugees in refugee settlements in Uganda, March 23, 2020 to March 31, 2021

	**Nationals**	**Refugees**	**Total**	***p* value**
Total number of cases	728 (N (%))	271 (N (%))	999 (N (%))	
Sex distribution				0.79
Female cases	313 (43.0)	119 (43.9)	432 (43.2)	
Male cases	415 (57.0)	152 (56.1)	567 (56.8)	
Age – mean (SD)	28.4 (14.6)	26.8 (14.7)	27.9 (14.6)	0.12
Most affected age groups	18–29 253 (34.9)	18–29 92 (33.9)	18–29 345 (34.6)	0.61
Regional distribution				< 0.01
Center	447 (61.4)	88 (32.5)	535 (53.6)	
South	62 (8.5)	107 (39.5)	169 (16.9)	
West Nile	219 (30.1)	76 (28.0)	295 (29.5)	

Sources of Data: UNHCR line list from refugee settlements

Data on symptoms (cough, body pain, fever, other flu-like symptoms) are available for 132 cases only (13.2% of the total line list). Of these, 30 (22.7%) reported symptoms. Data on comorbidities are available for 633 cases (63.4%). Only five cases reported having comorbidities: two females and three males; two nationals and three refugees; two cases in the older age group (60 + years) and the other three in the age groups (years) 12–17; 18–29; and 40–49. Two cases reported having diabetes, one asthma, one hypertension and one neurological conditions.

Table 4 shows the proportion of cases hospitalized, isolated, admitted to the intensive care unit (ICU) and by disease outcome. Data on hospitalization are available for 618 (61.9%) of the cases; on isolation for 601 (60.2%); on admission to ICU for 632 (63.3%) of the cases; and on disease outcomes for 942 (94.4%) of cases.

**Table 4:** Proportion of cases in refugee settlements by case management, sex, age, disease outcome and displacement status, Uganda (March 23, 2020 to March 31, 2021)

	**Hospitalized**	***p*-value**	**Isolation—Home**	**Isolation -Institution**	***p*-value**	**Intensive care unit**	***p*-value**	**Died**	**Recovered**	***p*- value**
**Available Data**										
N (% available of total line list)	618 (61.9)		601 (60.2)	601 (60.2)		632 (63.3)		7 (< 0.01)	935 (93.6%)	
Nationals	508 (50.9)		508 (50.9)	508 (50.9)		522 (52.3		4 (< 0.01)	686 (68.7%)	
Refugees	110 (11.0)		93 (9.3)	93 (9.3)		110 (11.0)		3 (< 0.01)	249 (24.9%)	
**From Data Available**										
Total (N (% of available data))	292 (47.2)		455 (75.7)	146 (24.3)		3 (0.5)		7 (0.7)	935 (99.3%)	
Nationals	197 (38.7)		411 (80.9)	97 (19.1)		1 (0.2)		4 (0.6)	686 (99.4%)	
Refugees	95 (86.4)		44 (47.3)	49 (52.7)		2 (1.2)		3 (1.1)	249 (91.9%)	
**Displacement Status** (N (% of available data))		** < 0.01**			** < 0.01**		**0.02**			0.33
Nationals	197 (67.5)		411 (90.3)	97 (66.4)		1 (33.3)		4 (57.1)	686 (73.4%)	
Refugees	95 (32.5)		44 (9.7)	49 (33.6)		2 (66.7)		3 (42.9)	249 (26.6%)	
**Sex** (N (% of available data))		0.51			0.44		0.73			0.48
Females	123 (42.1)		189 (41.5)	66 (45.2)		1 (33.3)		4 (57.1)	411 (44.0%)	
Males	169 (57.9)		266 (58.5)	80 (54.8)		2 (66.7)		3 (42.9)	524 (56.0%)	
**Age Group** (N (% of available data))		** < 0.01**			**0.02**		0.55			** < 0.01**
0–4	6 (2.1)		33 (7.3)	6 (4.1)		0 (0.0)		0 (0.0)	46 (4.9%)	
5–11	22 (7.6)		47 (10.4)	14 (9.7)		0 (0.0)		0 (0.0)	79 (8.5%)	
12–17	19 (6.6)		42 (9.3)	12 (8.3)		1 (33.3)		1 (14.3)	74 (7.9%)	
18–29	125 (43.3)		137 (30.2)	67 (46.2)		1 (33.3)		2 (28.6)	322 (34.5%)	
30–39	72 (24.9)		113 (24.9)	23 (15.9)		0 (0.0)		0 (0.0)	237 (25.4%)	
40–49	26 (9.0)		34 (7.5)	13 (9.0)		1 (33.3)		1 (14.3)	93 (10.0%)	
50–59	13 (4.5)		36 (7.9)	6 (4.1)		0 (0.0)		0 (0.0)	59 (6.3%)	
60 +	6 (2.1)		11 (2.4)	4 (2.8)		0 (0.0)		3 (42.9)	24 (2.6%)	
**Region** (N (% available data))		** < 0.01**			** < 0.01**		**0.04**			**0.02**
Center	221 (75.7)		388 (85.3)	143 (97.9)		1 (33.3)		2 (28.6)	527 (56.4%)	
South	18 (6.2)		0 (0.0)	1 (0.7)		1 (33.3)		0 (0.0)	163 (17.4%)	
West Nile	53 (18.2)		67 (14.7)	2 (1.4)		1 (33.3)		5 (71.4)	245 (26.2%)	

Sources of Data: UNHCR line list from refugee settlements

Almost half of the cases where information is available 292 (47.2%), required hospitalization; mainly among nationals (*N* = 197 (67.5%), men (*N* = 169 (57.9%), age group 18–29 years (*N* = 125 (43.3%), and in the Center region (*N* = 221 (75.7%)). Hospitalization rates differed by regions, ranging from 39.5 hospitalizations/100,000 population, 95%CI [31.48–49.60] in the Center region, to 0.62 hospitalizations/100,000 population, 95%CI [0.27–1.46] in West Nile.

Seven deaths were reported (four nationals and three refugees) out of the 999 cases, with a total case fatality ratio (CFR) of 0.7%. CFR among nationals was 0.6% and 1.1% among refugees. Deaths are similar by gender (men (3) and women (4)), and mainly occur in the West Nile region (five). Three of the deaths are in the older age group, while the other four occur in the age groups 12–17 years (1), 18–29 years (2) and 40–49 years (1).

Table 5 describes the risk factors for selected outcomes. Younger age is a protective factor against hospitalization. Older age is a risk factor for death, and being a refugee is a risk factor for hospitalization and isolation in an institution (compared to remaining at home). We did not observe any differences in outcomes by sex.

**Table 5:** Adjusted odds ratios for disease management and outcome in refugee settlements, March 23, 2020 to March 31, 2022

	**Hospitalization [95%CI]**	***p*-value**	**Isolation [95%CI]**	***p*-value**	**ICU [95%CI]**	***p*-value**	**Died [95%CI]**	***p*-value**
Male Sex	1.17 [0.82—1.67]	0.40	0.90 [0.60—1.33]	0.59	1.67 [0.15- 18.81]	0.68	0.77 [0.16—3.68]	0.74
Age 0–17	**0.28 [0.18—0.44]**	** < 0.001**	0.77 [0.48 – 1.23]	0.27	1.40 [0.12—15.81]	0.79	1.08 [0.11 – 10.50]	0.95
Age 60 +	0.506 [0.17—1.51]	0.22	1.42 [0.43 – 4.63]	0.56	0	-	**33.29 [6.05 – 183.06]**	** < 0.001**
Refugee	**12.30 [6.73–22.47]**	** < 0.001**	**4.80 [3.01 – 7.65]**	** < 0.001**	9.68 [0.87 – 108.11]	0.07	2.60 [0.54—12.54]	0.24

Sources of Data: UNHCR line list from refugee settlements

References categories are: female, age group 18–59 years and nationals

### Impact on routine health services and other health outcomes

**Fig. 2:**
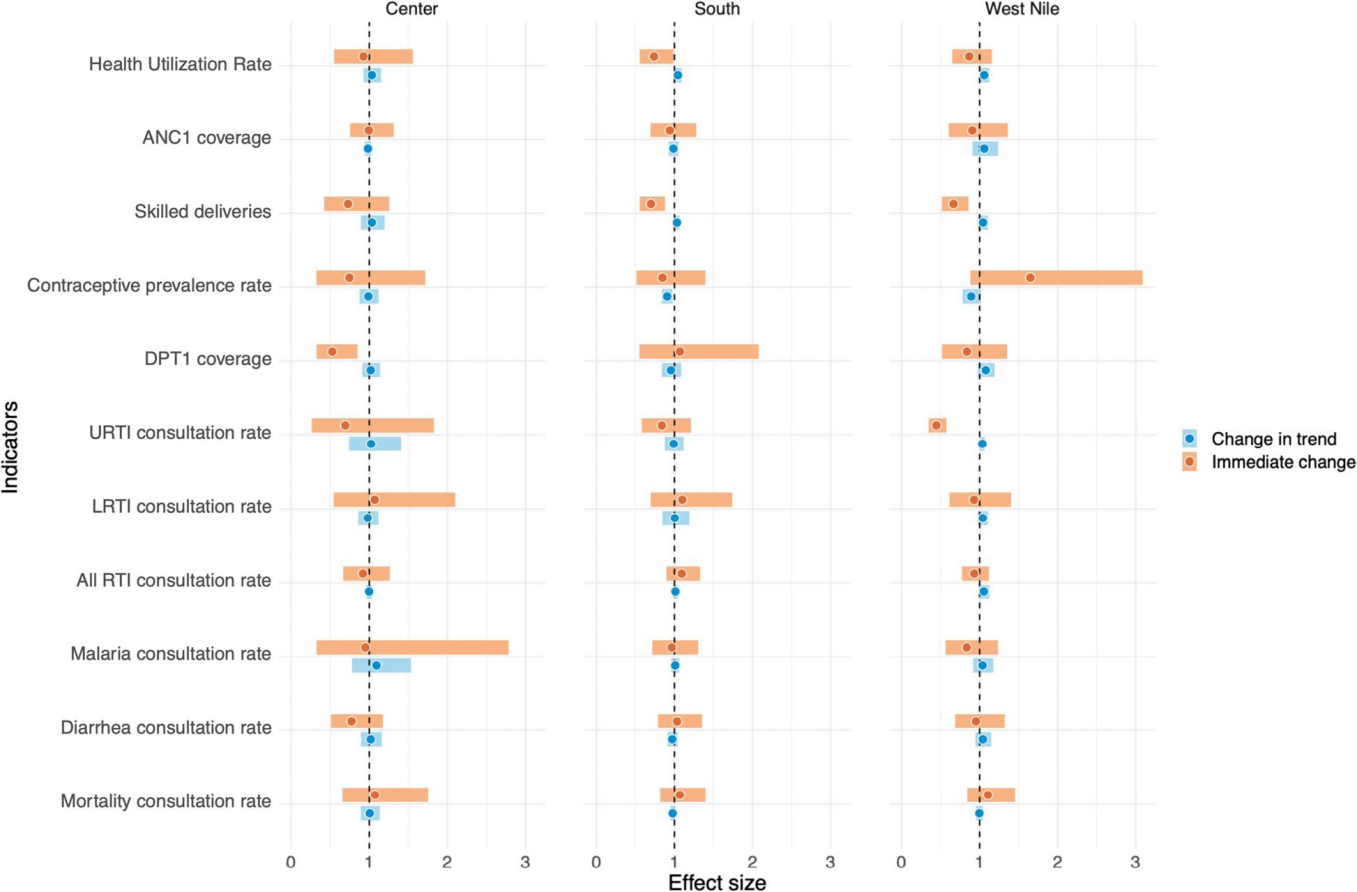
Overview of interrupted time series results on health services and health outcomes in refugee settlements in Uganda, by region, January 1, 2017 to March 31, 2021. Note: the dot indicates the coefficient estimate and the bar the confidence intervals. CIs encompassing 1 (dotted vertical line) indicate results that are not statistically significant. Acronyms: ANC1: first antenatal care visit; DPT1: first dose of diphtheria, pertussis, and tetanus vaccine; URTI: Upper respiratory tract infection; LRTI: Lower respiratory tract infection; RTI: Respiratory tract infection

Overview of interrupted time series results are in Table 6 and Fig. 2 below.

**Table 6:** Interrupted Time Series results: impact of COVID-19 on routine health services and health outcomes in refugee settlements in Uganda, by region, January 1, 2017 – March 31, 2021

	West Nile				Center				South			
	IRR^a^ immediate effect [95% CI^b^]	*p*-value	IRR^a^ change in trend [95% CI^b^]	*p*-value	IRR^a^ immediate effect [95% CI^b^]	*p*-value	IRR^a^ change in trend [95% CI^b^]	*p*-value	IRR^a^ immediate effect [95% CI^b^]	*p*-value	IRRa change in trend [95% CIb]	*p*-value
Health utilization rate	0.953 [0.687 – 1.322]	0.774	1.042 [0.947 – 1.147]	0.394	0.773 [0.509 – 1.175]	0.228	1.019 [0.894 – 1.161]	0.780	1.033 [0.789 – 1.352]	0.814	0.972 [0.910 – 1.039]	0.405
Mortality rate	1.649 [0.881 – 3.088]	0.118	0.891 [0.781 – 1.016]	0.086	0.746 [0.324 – 1.717]	0.490	0.989 [0.875 – 1.119]	0.866	0.847 [0.514 – 1.396]	0.515	**0.906 [0.842 – 0.975]**	**0.009**
*Maternal and reproductive health*												
ANC1^c^ coverage	1.106 [0.842 – 1.453]	0.469	0.996 [0.952 – 1.042]	0.854	1.074 [0.657 – 1.754]	0.776	1.006 [0.892 – 1.134]	0.926	1.069 [0.817 – 1.397]	0.627	0.977 [0.946 – 1.010]	0.175
Skilled delivery coverage	0.907 [0.605 – 1.360]	0.636	1.060 [0.908 – 1.237]	0.460	0.995 [0.754 – 1.313]	0.970	0.983 [0.944 – 1.025]	0.428	0.941 [0.693 – 1.278]	0.697	0.986 [0.925 – 1.052]	0.678
Contraceptive prevalence	0.929 [0.615 – 1.403]	0.725	1.040 [0.975 – 1.109]	0.234	1.070 [0.545 – 2.100]	0.845	0.980 [0.860 – 1.117]	0.762	1.100 [0.696 – 1.739]	0.682	1.004 [0.846 – 1.191]	0.966
*Child Health*												
DPT1^d^ Vaccination coverage	0.931 [0.774 – 1.120]	0.449	1.054 [0.987 – 1.126]	0.117	0.919 [0.667 – 1.265]	0.604	0.998 [0.960 – 1.038]	0.930	1.092 [0.898 – 1.328]	0.377	1.012 [0.982 – 1.042]	0.447
*Infectious diseases*												
URTI^e^ rate	0.867 [0.650 – 1.157]	0.332	1.059 [0.999 – 1.122]	0.052	0.926 [0.550 – 1.558]	0.771	1.033 [0.927 – 1.151]	0.560	**0.739 [0.554 – 0.988]**	**0.041**	1.044 [0.997 – 1.094]	0.068
LRTI^f^ rate	**0.448 [0.348 – 0.575]**	** < 0.001**	**1.035 [1.003 – 1.067]**	**0.031**	0.694 [0.264 – 1.828]	0.460	1.023 [0.742 – 1.408]	0.891	0.839 [0.581 – 1.211]	0.348	0.990 [0.877 – 1.117]	0.867
All RTI^g^ rate	**0.666 [0.517 – 0.858]**	**0.002**	1.044 [0.983 – 1.108]	0.163	0.729 [0.424 – 1.256]	0.255	1.034 [0.893 – 1.197]	0.655	**0.699 [0.555 – 0.880]**	**0.002**	**1.032 [1.000 – 1.065]**	**0.050**
Malaria rate	0.838 [0.519 – 1.352]	0.469	1.079 [0.977 – 1.191]	0.133	**0.527 [0.326 – 0.851]**	**0.009**	1.019 [0.911 – 1.140]	0.743	1.070 [0.551 – 2.080]	0.842	0.955 [0.839 – 1.086]	0.481
Diarrhea rate	0.835 [0.564 – 1.236]	0.368	1.038 [0.917 – 1.176]	0.555	0.951 [0.325 – 2.784]	0.927	1.093 [0.779 – 1.534]	0.606	0.966 [0.716 – 1.303]	0.821	1.009 [0.961 – 1.060]	0.709

Bold results indicate a statistically significant result (confidence interval does not include 1)

Acronyms: ^a^ *IRR* Incidence rate ratio, ^b^ *CI* Confidence interval, ^c^ *ANC1* First antenatal care visit, ^d^ *DPT1* First dose of diphtheria, pertussis, and tetanus vaccine, ^e^ *URTI* Upper respiratory tract infection, ^f^ *LRTI* Lower respiratory tract infection, ^g^ *RTI* Respiratory tract infection

#### Health care utilization rate

**Fig. 3:**
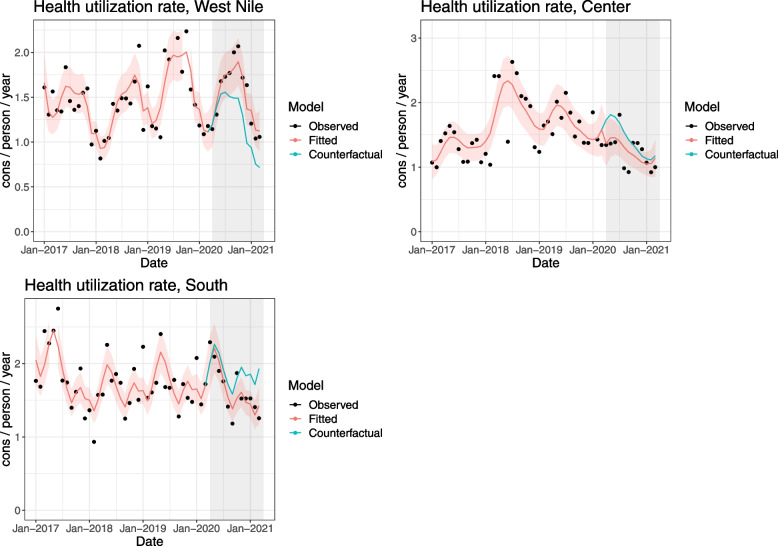
Interrupted time series of health care utilization rate among refugees (expressed as consultations/ person / year) in refugee settlements in Uganda, by region, 2017–2021

No major changes can be seen in rates of outpatient consultations, neither at the beginning of the pandemic (immediate effect) nor during the COVID-19 period (change in trend) (Fig. 3). The Center region shows a 23% decrease at the beginning of the pandemic, which however seems in line with the decreasing trend since early 2018. We cannot link the drop with COVID-19. The trend during the COVID-19 period does not seem to differ from the pre-COVID-19 period.

#### Maternal and reproductive health

Even in the pre-COVID-19 period, values of ANC1 coverage are highly variable and exceed 100% for many of the months in all three regions (Figure S2 Supplementary materialfile). In both South and Center, ANC1 coverage is on average over 200% and reaches over 500% in Center. All estimates are not statistically significant, pointing to insufficient evidence to conclude that ANC1 services were affected.

Lagged results for coverage of skilled deliveries are presented for the Center (2-months) and South (3-months) regions (see sensitivity analysis in Supplementary material). No immediate change is observed and trends during the COVID-19 period do not seem to differ from the pre-COVID-19 period (all coefficients are not statistically significant).

Unlike the other two regions, West Nile reports an increasing trend in contraceptive prevalence since 2017, which continues uninterrupted by the pandemic (4-month lagged results – see sensitivity analysis in Supplementary material). The Center and South regions show a decreasing trend from 2017; this seems to be interrupted in the Center region where a small immediate increase can be seen 5 months after the beginning of the pandemic (5-month lagged results IRR: 1.070, 95%CI [0.545 – 2.100]), however trends during COVID-19 period do not differ from pre-COVID-19. An immediate small increase and a slightly increasing slope are also observed in the South, however, the results are not statistically significant.

#### Child immunization

DPT1 Immunization services do not seem to be particularly affected at the beginning and during the COVID-19 pandemic (Figure S3 Supplementary material). Vaccination coverage in West Nile was below 80% and decreasing since early 2019; this decreasing trend seems to be interrupted at the beginning of the pandemic, but results cannot be associated with COVID-19. Coverage in the Center region was around 100% and while a small decrease can be seen (-8%), confidence intervals are large and results not significant. One-month lagged results are reported for the South region where the decreasing trend since 2017 seems to be interrupted at the beginning of the pandemic. Results are however not statistically significant.

#### Respiratory tract infections

**Fig. 4:**
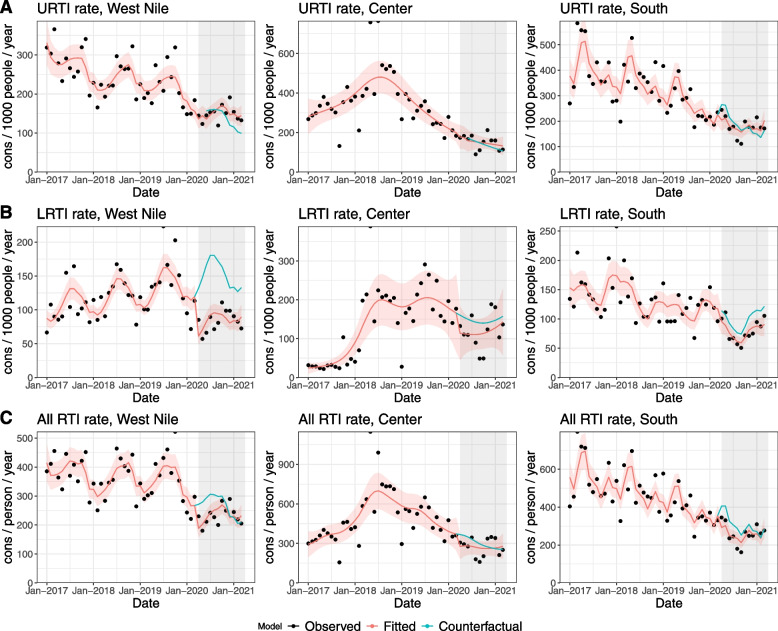
Interrupted time series results for Respiratory Tract Infections by type: **A** Upper respiratory tract infections (URTI); **B** Lower respiratory tract infections (LRTI); **C** All respiratory tract infections (RTI) in refugee settlements by region, Uganda, 2017–2021

Estimates for all RTI, as well as disaggregated by upper and lower tract infections, show an immediate drop at the beginning of the pandemic in the three regions (Fig. 4). Decreases for all RTI consultations include 33% in West Nile (IRR: 0.666, 95%CI [0.517 – 0.858]), 30% in South region (IRR: 0.699, 95%CI [0.555–0.880], and 27% in Center (IRR: 0.729, 95%CI [0.424 – 1.256]). Decrease in LRTI was higher in West Nile (by 55%, IRR: 0.448, 95%CI [0.348 – 0.575] than in Center (by 31%) and in South (by 16%, 1-month lagged results). Results are not significant in the last two regions. Upper respiratory tract infections seem less affected, except for South region where a 26% decrease can be observed (IRR: 0.739, 95%CI [0.554 – 0.988]). Lagged results for West Nile and Center are reported (both 3-month lag). Change in slopes was positive in the three regions for the three indicators, showing some catching up during the COVID-19 months.

#### Infectious diseases

A drop in consultations for malaria is noted mainly in Center region (by 47%, IRR: 0.527, 95%CI [0.326 – 0.851]) (Figure S4 Supplementary material). No clear change can be seen in either West Nile (3-month lagged results, see Supplementary material for sensitivity analysis), or South regions. Consultations for diarrhea do not seem to be affected (Figure S4 Supplementary material). While small decreases are reported in the three regions, they do not seem to differ from previous decreasing trends.

#### Mortality

An absolute number of deaths in all settlements over the entire study period is low; less than 4,400 deaths across all settlements over the study period (corresponding to 8.4 / 10,000/ year). Consequently, results should be interpreted with caution. Because of sparse data, Interrupted Time Series analysis is conducted only on crude mortality rates (CMR; all ages, all causes). The CMRs show an immediate drop at the beginning of the pandemic in the Center and South (not significant) (Figure S5 Supplementary material). West Nile reported a 65% increase (also not significant). All regions report a decreasing slope, which is statistically significant in the South (IRR: 0.906, 95%CI [0.842 – 0.975]).

## Discussion

The first COVID-19 case among refugees was registered on May 22, 2020, in the Adjumani settlement, two months after the first case was reported in Uganda. The refugee settlements and regions experienced peaks at different points in time, with West Nile reporting a peak around June 2020, Center reporting two peaks around September and October 2020, and South reporting two peaks around November and December 2020. West Nile’s peak, while earlier than the other regions, was smaller than the following waves in the Center and South regions (Fig. 1). Compared to the national epi curve (Fig. 1), the first peak in West Nile in June 2020 occurred earlier, while the other peaks appear to be in line with national trends, yet at a smaller magnitude. Since COVID-19 data on cases in Uganda are not disaggregated by district, we are, unfortunately, not able to compare trends in settlements to those of host communities within the same district.

Incidence rates (Table 2) were higher at the national level for the general population compared to refugees by region and overall. However, this could reflect a lower testing capacity in the settlements compared to the national level (Table 2). We cannot compare testing or incidence rates among refugees to nationals in and around the settlements, as there are no national population estimates in that localized geographic area (Fig. 1). The percent positive was very different among the three regions: South (13.5%) was higher than the national average (8.1%), Center was similar (7.3%), and West Nile was much lower (1.5%; Table 6). It is not possible to know whether varying percent positive reflect different stages of testing strategies, different population susceptibility, or differences in the availability of tests, since testing data are unavailable per month. Possible factors contributing to higher percent positive in the South could include lower testing capacity and the presence of more congregated settings (than in other regions) which could facilitate transmission.

From the limited available data, COVID-19 cases among refugees in Uganda seem to align with the global epidemiology of COVID-19 [32]. The majority of cases were among men, mainly in the middle age groups (18–29 years and 30–39 years), with a case fatality rate of 1.1%. Persons 60 + years were confirmed at higher risk for fatal outcomes, and younger age was a protective factor against hospitalization. Notably, the proportion of hospitalized cases in the settlements (Table 4) was much higher than that seen globally (4–10%) [32]; 35% of total refugee cases were hospitalized, corresponding to 86% of refugee cases with available data. The majority of the hospitalizations occurred in the Central region (75%). Differences in hospitalization among regions were due to a variety of reasons, including different capacity to implement home-based treatment and varying availability of treatment units. Furthermore, at the beginning of the epidemic, Uganda’s national strategy was to hospitalize all persons who were COVID-19 positive, regardless of the severity. This strategy contributed to very high admission rates. As the Ugandan policy changed towards home-based care for non-severe cases, the hospitalization rates declined. Unfortunately, the hospitalization dates are mostly not available, limiting the analysis of their evolution over time.

The COVID-19 data have limitations. First, different line list templates were used across settlements, leading to only a few variables being consistently collected across settlements. Consequently, the completeness of several variables is low, and, therefore, results may not reflect the overall COVID-19 cases among refugees. A harmonized line list across settlements was introduced in June 2021. Second, the recording of dates was problematic. Different dates were recorded across settlements (i.e., date of sample collection, date of the test, date of tests results); however, the three dates were not collected for the same case, making it difficult to analyze the turnaround for different testing steps and to report in a consistent manner. Furthermore, dates were not consistently recorded in the same date format, which resulted in complications while trying to rectify the dates during analysis. Finally, there was no clear system in place to obtain dates from various data sources (e.g., laboratory for testing, health facilities for hospitalization, or individuals for symptom onset), which could have increased completeness. Extensive cleaning before and during analysis and additional checking of dates were performed. UNHCR public health officers were asked to verify entries, which improved the data quality. However, not all inconsistencies were able to be clarified.

Investigating how and whether health services were affected by the COVID-19 pandemic provides useful insights into the health system’s resilience and capacity to maintain essential health services during shocks. The indirect effects of COVID-19 on routine health services and outcomes appear quite consistent across regions. Routine and preventative health services seem to have been less affected by the COVID-19 pandemic than consultations for acute conditions related to infectious diseases.

Outpatient consultations, as well as maternal and reproductive health services, appear to have maintained pre-COVID-19 levels. These services continued to be provided and efforts were made to increase outreach in the communities, and to ensure that women could reach health facilities, even under travel restrictions. Maternal services were delivered as much as possible in health facilities within the settlements to be as close as possible to the communities. As travel restrictions applied to movements between districts, transfers within settlement were possible. Furthermore, pregnant women were given priority to access facilities through “boda boda” (i.e., motorbike taxis) or UNHCR partners’ ambulances [33]. Contraceptive coverage also did not appear to have been affected, likely because the majority of women prefer long-term methods such as injections and implants (UNHCR routine data and [34]). The drug distribution schedule was also increased: monthly contraceptive pills were given to cover three months, and an additional injection dose was given to reduce visits to health facilities. Child immunization services showed little to no changes due to COVID-19. Our findings on maternal and child health appear to deviate from the results of the only analysis (to our knowledge) that estimated the effects of COVID-19 on health service utilization at national level in Uganda [35]. In this study, an immediate reduction in antenatal care, deliveries and immunization services was reported at the beginning of the pandemic; however, services recovered rapidly by June 2020.

When examining acute conditions, consultations for RTIs showed an immediate drop at the beginning of the pandemic. This decrease could relate to the positive externalities of the public health and social measures implemented to limit the spread of COVID-19 (e.g., mask use and hand washing as well as reduced interactions), which may have reduced non-COVID-19 respiratory infections as was observed in other countries [36, 37]. However, it could also indicate a change in healthcare-seeking behavior that led to reduced care for RTIs, possibly due to fear of being tested and isolated or quarantined due to COVID-19 [38, 39]. Over time, this fear may have faded due to increased information, awareness campaigns and sensitization activities, which seems to be reflected in the catch-up that is noted in the results. Consultations for both malaria and diarrhea were reduced at the beginning of the pandemic, which is similar to what was found at the national level [35]. However, a study of malaria surveillance data from 17 reference centers in rural Uganda did not find any difference between observed and expected outpatient visits, or malaria cases [40]. The drop we observed may have been due to fewer people seeking health care because of mobility restrictions or avoiding health care facilities due to concerns about a COVID-19 diagnosis and subsequent measures.

Finally, no clear effect on mortality was observed at the beginning of the pandemic, although a decreasing trend was identified. Given the relatively few COVID-19 deaths that occurred in this population during the study period, estimating the expected values and counterfactuals is challenging. UNHCR’s mortality reporting system currently triangulates health facility data with community reports to ensure that most deaths are captured, including those in the community. Mortality should be further monitored in the second year of the pandemic to better understand longer-term trends.

Conducting Interrupted Times Series analyses in volatile settings such as refugee settlements can be challenging, as it may be difficult to meet some analytical assumptions, and mitigate threats to validity. First, considering the COVID-19 period as one homogenous period likely does not capture changes in transmission patterns, non-pharmaceutical interventions, policies, perceptions and behaviors. This is therefore a simplification of reality. Second, factors such as new population arrivals, decisions to open/close settlements, policy changes, or increased/decreased funding to different types of services, as well as outbreaks, made it difficult to identify a “normal” period and establish a pre-COVID-19 comparison. As discussed above, selected months during the study period showed erratic values in several settlements (even after aggregation at regional level), which reduced the model fit and limited the capacity of the analysis to identify statistically significant changes. Third, seasonality may have been a time-varying confounder that varied over years, and the autocorrelation structure of order 1 used in the analysis may not adequately capture autocorrelation. Other confounders or effect modifiers may have been important but are not considered in the model. Fourth, limitations related to UNHCR’s HIS data include possible disparities in data quality, timeliness and completeness across settlements given the multiplicity of reporting partners. However, tools and formats have been standardized to increase quality and UNHCR provides regular training. As routine health data reflect health seeking-behavior, we were not able to estimate how the incidence of diseases in the population has changed during the pandemic. Fifth, conducting analysis at regional level may have masked heterogeneity or differential COVID-19 effects among the settlements. Finally, the lack of an estimate of the host population using health services in settlements limited the analysis and comparisons which we could conduct.

The COVID-19 pandemic also had important socio-economic and psychological effects, specifically on refugee populations, that were not captured by our analysis. The third round of the high-frequency phone survey for refugees in Uganda (Feb/March 2021) [41] reported that there was still a high level of food insecurity, difficulty in accessing medicines, and an increase in self-reported depression, particularly among women, elderly and those living in the West Nile region. Furthermore, refugees had limited opportunities to access savings or borrow money to face emergencies. The same phone survey found that refugees in Uganda may have been more negatively affected by COVID-19 than nationals, and had a lower capacity to recover over time. However, another assessment of the COVID-19 impact on livelihoods of both host communities and refugees in the West Nile and South regions reported important reductions in monthly income in both groups [42].

Furthermore, the situation for urban refugees living in Kampala might have been quite different, as they receive less direct support than refugees living in officially demarcated settlements, where services and protection are provided by UNHCR and partners [43]. For example, UNHCR had to contract private or semi-private hospitals that are accessible to urban refugees and set up an ambulance service to pick up patients from home. Cash grants were also introduced to support economically urban refugees. This highlights the vulnerability of refugee populations even in welcoming settings like Uganda, where both socio-economic and health impacts may have been exacerbated due to a combination of factors such as reduced social support, limited access to services and information in appropriate language, and reduced economic opportunities [43]. A gender-sensitive analysis [44] showed that women and girls were disproportionately affected by COVID-19 through additional unpaid work, reduced income, and increased risk for negative coping mechanisms. In addition, lost schooling will have long-term impacts that are more difficult to estimate at this time. Finally, the findings from refugees in Ugandan settlements cannot be generalized to other refugee settings with very different conditions. For example, infection rates in crowded reception facilities on Greek islands were higher than among the general Greek population [45]. Ensuring appropriate living conditions and access to water and sanitation, and health services remain central to reducing the risk of infection among refugees.

## Conclusions

We estimate the direct and indirect effects of the COVID-19 pandemic in Ugandan refugee settlements during the first year of the pandemic. Transmission in the settlements appears to be lower than in Uganda; however testing rates were also lower. Many mild or asymptomatic cases were likely missed. Routine and preventative health services appear to have been little affected by the COVID-19 pandemic, while immediate reductions were reported mostly for infectious disease consultations. The situation may have been very different in the second and third years of the pandemic, with more contagious variants. This study calls for future research investigating how infection susceptibility has changed over time and which response and containment strategies have successfully contributed to maintaining health services. These lessons will inform preparedness and response strategies for future pandemics.

## Supplementary Information

**Additional file 1:** Supplementary material about methods and results. **Figure S1.** Map of refugee settlements in Uganda (UNHCR). **Figure S2.** Interrupted time series for selected sexual and reproductive health services (A: first visit of antenatal care, B: skilled deliveries, and C: contraceptive prevalence rate) in refugee settlements by region, Uganda, 2017-2021 (2018-2021 for deliveries). **Figure S3.** Interrupted time series results for coverage of DPT1 vaccine in refugee settlements, by region, Uganda, 2017-2021. **Figure S4.** Interrupted time series results for infectious diseases consultations in refugee settlements: malaria (A) and diarrhea (B), by region, Uganda 2017-2021. **Figure S5.** Interrupted time series results for crude mortality rate in refugee settlements by region, Uganda 2017-2021. **Table S1.** Definition of outcome indicators included in the Interrupted Time Series analysis. **Table S2.** Model specification for Interrupted Time Series analysis. **Table S3.** Completeness of variables included in the aggregated COVID-19 line list, Uganda refugee settlements.

## Data Availability

The datasets generated and/or analyzed during the current study are not publicly available due to the possible vulnerability of the study population but are available from the corresponding author on reasonable request pending approval of UNHCR.

## Abbreviations

ANC1: First visit of antenatal care
BDI: Burundi
CFR: Case Fatality Rate
CI: Confidence Interval
CMR: Crude Mortality Rate
DPT1: First dose of diphtheria, pertussis and tetanus vaccine
DRC: Democratic Republic of the Congo
HIS: Health Information System
ICU: Intensive Care Unit
IRR: Incidence Rate Ratio
ITS: Interrupted Time Series
LRTI: Low Respiratory Tract Infections
RTI: Respiratory Tract Infections
RWA: Rwanda
SD: Standard Deviation
SOM: Somalia
SSD: South Sudan
UNHCR: United Nations High Commissioner for Refugees
URTI: Upper Respiratory Tract Infections
