# Supplementary material for: COVID-19 epidemiology and changes in health service utilization in Uganda’s refugee settlements during the first year of the pandemic
Source: BMC Public Health. 2022 Oct 17;22:1927. doi: 10.1186/s12889-022-14305-3 (PMC9574818; doi:10.1186/s12889-022-14305-3)
Supplement: Supplementary file 1 — Additional file 1: Supplementary material about methods and results. Figure S1. Map of refugee settlements in Uganda (UNHCR). Figure S2. Interrupted time series for selected sexual and reproductive health services (A: first visit of antenatal care, B: skilled deliveries, and C: contraceptive prevalence rate) in refugee settlements by region, Uganda, 2017-2021 (2018-2021 for deliveries). Figure S3. Interrupted time series results for coverage of DPT1 vaccine in refugee settlements, by region, Uganda, 2017-2021. Figure S4. Interrupted time series results for infectious diseases consultations in refugee settlements: malaria (A) and diarrhea (B), by region, Uganda 2017-2021. Figure S5. Interrupted time series results for crude mortality rate in refugee settlements by region, Uganda 2017-2021. Table S1. Definition of outcome indicators included in the Interrupted Time Series analysis. Table S2. Model specification for Interrupted Time Series analysis. Table S3. Completeness of variables included in the aggregated COVID-19 line list, Uganda refugee settlements. [file 12889_2022_14305_MOESM1_ESM.docx]

**Supplementary material**

Table of Contents

[1 Methods 1](#_Toc112833723)

[1.1 Study setting 1](#_Toc112833724)

[1.2 Definitions of study outcomes 4](#_Toc112833725)

[1.3 Interrupted Time Series – model specifications 4](#_Toc112833726)

[2 Additional Results 5](#_Toc112833727)

[2.1 Completeness of dataset by variable 5](#_Toc112833728)

[2.2 Figures 6](#_Toc112833729)

[2.3 Model fit check, by variable 9](#_Toc112833730)

[2.3.1 Health utilization rate 9](#_Toc112833731)

[2.3.2 ANC1 10](#_Toc112833732)

[2.3.3 Skilled deliveries 12](#_Toc112833733)

[2.3.4 Contraceptive prevalence 12](#_Toc112833734)

[2.3.5 DPT1 13](#_Toc112833735)

[2.3.6 URTI 14](#_Toc112833736)

[2.3.7 LRTI 14](#_Toc112833737)

[2.3.8 All RTI 15](#_Toc112833738)

[2.3.9 Malaria 15](#_Toc112833739)

[2.3.10 Diarrhea 16](#_Toc112833740)

[2.3.11 Mortality 17](#_Toc112833741)

# Methods

## Study setting

Figure S1: Map of refugee settlements in Uganda (UNHCR)

### COVID-19 measures implemented in the refugee settlements

**Coordination** - Support to coordination at national and subnational levels – This started in March 2020 even before Uganda had registered the first case. It was extended to all districts and all settlements created task force committees to oversee the implementation of covid prevention measures and preparations for case management. This is still in place in all settlements at the time of writing the paper (August 22). At national level a national task force chaired by the Prime minister was established to coordinate the response across six pillars (governance and leadership; surveillance and laboratory; case management; logistics; risk communication, social mobilization and community engagement; mental health psychosocial support). At district level the District Health officer chaired the district task force. UNHCR public health officers at national and settlement level participated to the task force.

**Surveillance** - Health facility, institutional and community surveillance through the village health teams, social workers, protection workers and teachers. Surveillance was introduced by January 2020 and implemented in the communities, at facilities, social gatherings, at schools. It is ongoing in all settlements. Refugee leaders in the community were also responsible for informing the health partner of any new arrivals within the settlement who had evaded the normal entry and registration procedures so that they could be screened in accordance with the new arrival criteria.

**Risk communication** – With the community-based protection team messages were developed based on the MoH SBCC messaging for COVID and translated into local languages. Boda boda talk-talk, door-to-door, posters and peer-to-peer messaging were also used to sensitize the community. The “Feedback Referral and Resolution Mechanism” (FRRM), an already existing hotline providing information and referrals, was also used to provide information on COVID 19. – This started in January 2020 and is still active in all settlements.

**IPC -** Distribution of masks for refugees in the settlements, provision of hand sanitizers in communal institutions, health facilities, provision of soap and handwashing facilities in the refugee settlements – This started in March 2020 and is still ongoing in all settlements.

**Containment** – Contacts of positive case or new arrivals were moved in the institutional quarantine across the operation to reduce infection rates – This started in March 2020 in all settlements and is ongoing. However, the institutional quarantine has been replaced with home isolation.

**Case management** – All positive cases were moved into established treatment centers in the settlement and district hospitals - started in May 2020 and still ongoing.

**Psychosocial support** – Provided through the psychosocial partners for the patients in the treatment centers, on the ward and those in quarantine.

**Service continuation** – Provision of medicines for longer periods (multi-month dispensing ) for NCDs, ART, TB, use of VHTS to provide services in the communities, community outreaches in the communities

**Movement restrictions** – Stay at home order was effective between March 31, 2020 to June 2^nd^ 2020; movements, gatherings and public transports were banned.

## Definitions of study outcomes

Table S1: Definition of outcome indicators included in the Interrupted Time Series analysis.

| **Indicator name** | **Definition** | **Numerator** | **Denominator** |
| --- | --- | --- | --- |
| Health utilization rate | Average number of outpatient consultations per person per year | Number of outpatient consultations per month multiplied by 12 | Population |
| ANC1 coverage | Coverage of antenatal care | Number of first antenatal consultation per month multiplied by 12 | Expected number of pregnancies in population (estimated as 2.2% of the population) |
| Deliveries by skilled health workers | Proportion of deliveries attended by skilled health workers | Number of deliveries at health facilities attended by skilled health workers multiplied by 12 | Estimated number of deliveries in a year (based on crude birth rate, which varies by year) |
| Contraceptive prevalence rate |  | Number of family planning acceptors (new and repeat) multiplied by 12 | Number of women 15-49 years |
| Vaccination coverage DPT1 | Proportion of infants vaccinated with the first DPT dose | Number of infants (0-11 months) who received DPT1 vaccine per month multiplied by 12 | Estimated infant population (22% of the under 5 population) |
| Consultation rate for respiratory infections | Average number of consultations for respiratory infections per year per 1,000 | Number of consultations for respiratory infections per month multiplied by 1,000 by 12 | Estimated population |
| Consultation rate for malaria | Average number of consultations for malaria per year per 1,000 | Number of malaria consultations per month multiplied by 1,000 by 12 | Estimated population |
| Consultation rate for diarrheal diseases | Average number of consultations for diarrheal diseases per year per 1,000 | Number of diarrheal diseases consultations per month multiplied by 1,000 by 12 | Estimated population |
| Crude Mortality rate | Number of deaths per 1,000 in the total population per year | Number of deaths all causes, all ages per month multiplied by 1,000 by 12 | Estimated population |

## Interrupted Time Series – model specifications

Table S2: Model specification for Interrupted Time Series analysis.

|  | **Model specification** | | |
| --- | --- | --- | --- |
|  | Core predictors | Seasonality | Sensitivity analysis using lag |
| Health utilization rate | Yes | Included |  |
| Mortality rate | Yes | Included | Yes |
| LRTI | Yes | Included | Yes |
| URTI | Yes | Included | Yes |
| all RTI | Yes | Included | Yes |
| Diarrhea | Yes | Included | Yes |
| Malaria | Yes | Included |  |
| Contraceptive prevalence | Yes | Removed | Yes |
| ANC1 coverage | Yes | Removed | Yes |
| Skilled delivery coverage | Yes | Removed | Yes |
| Vaccination coverage | Yes | Removed | Yes |

Core predictors include population; Centered Month_i­_; COVID; Month since COVID_i_

# Additional Results

## Completeness of dataset by variable

Table S3: Completeness of variables included in the aggregated COVID-19 line list, Uganda refugee settlements

| **Data availability (N by variable)** | **Total** | | **Nationals** | | **Refugees** | |
| --- | --- | --- | --- | --- | --- | --- |
|  | **N=1001** | **%** | **N=728** | **%** | **N=271** | **%** |
| Age | 998 | 99.7% | 725 | 99.6% | 271 | 100% |
| Sex | 1001 | 100.0% | 728 | 100.0% | 271 | 100% |
| Date of sample collection | 830 | 82.9% | 677 | 93.0% | 152 | 56% |
| Date of test | 160 | 16.0% | 53 | 7.3% | 106 | 39% |
| Date of test results | 812 | 81.1% | 663 | 91.1% | 148 | 55% |
| Combined date (sample collection or date of test) | 975 | 97.4% | 716 | 98.4% | 257 | 95% |
| Presence of symptoms | 133 | 13.3% | 109 | 15.0% | 23 | 8% |
| Level of severity | 600 | 59.9% | 489 | 67.2% | 110 | 41% |
| Presence of comorbidities | 634 | 63.3% | 507 | 69.6% | 126 | 46% |
| Hospitalization / home treatment | 619 | 61.8% | 508 | 69.8% | 110 | 41% |
| Date of hospitalization | 277 | 27.7% | 183 | 25.1% | 93 | 34% |
| ICU / Oxygen | 633 | 63.2% | 522 | 71.7% | 117 | 43% |
| Date of discharge | 589 | 58.8% | 491 | 67.4% | 97 | 36% |
| Treatment outcome | 1001 | 100.0% | 728 | 100.0% | 271 | 100% |

## Figures


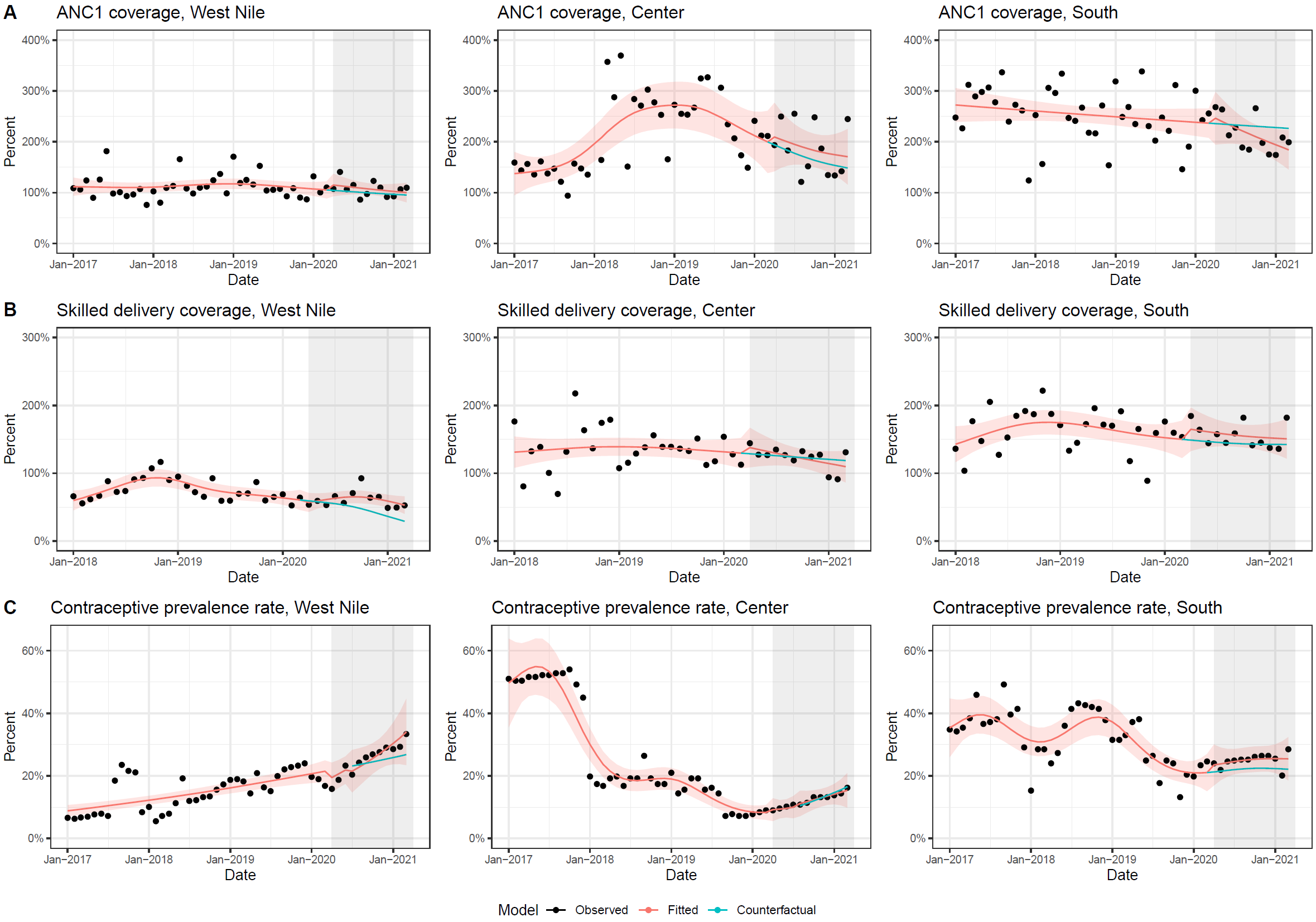


Figure S2: Interrupted time series for selected sexual and reproductive health services (A: first visit of antenatal care, B: skilled deliveries, and C: contraceptive prevalence rate) in refugee settlements by region, Uganda, 2017-2021 (2018-2021 for deliveries)


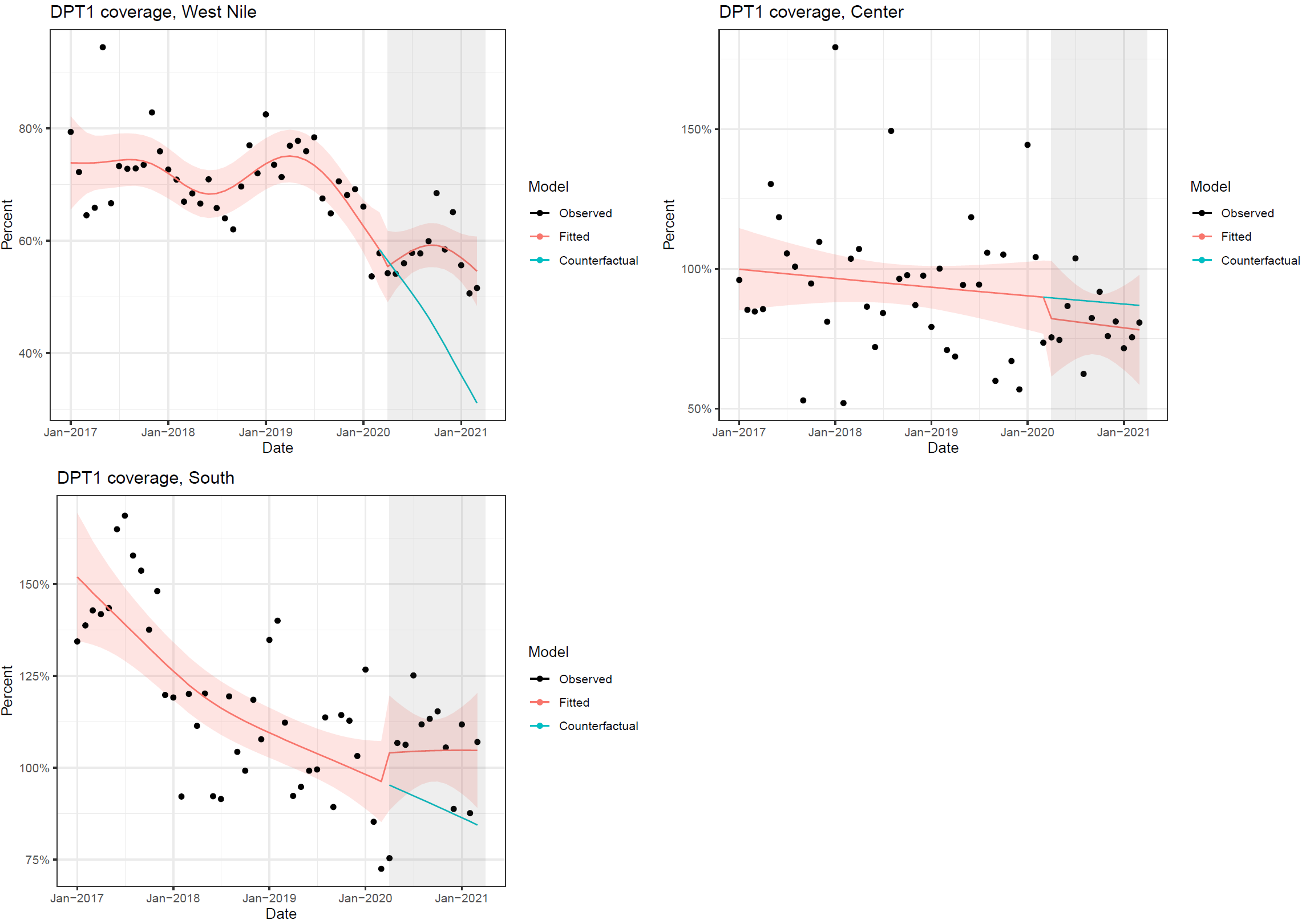


Figure S3: Interrupted time series results for coverage of DPT1 vaccine in refugee settlements, by region, Uganda, 2017-2021


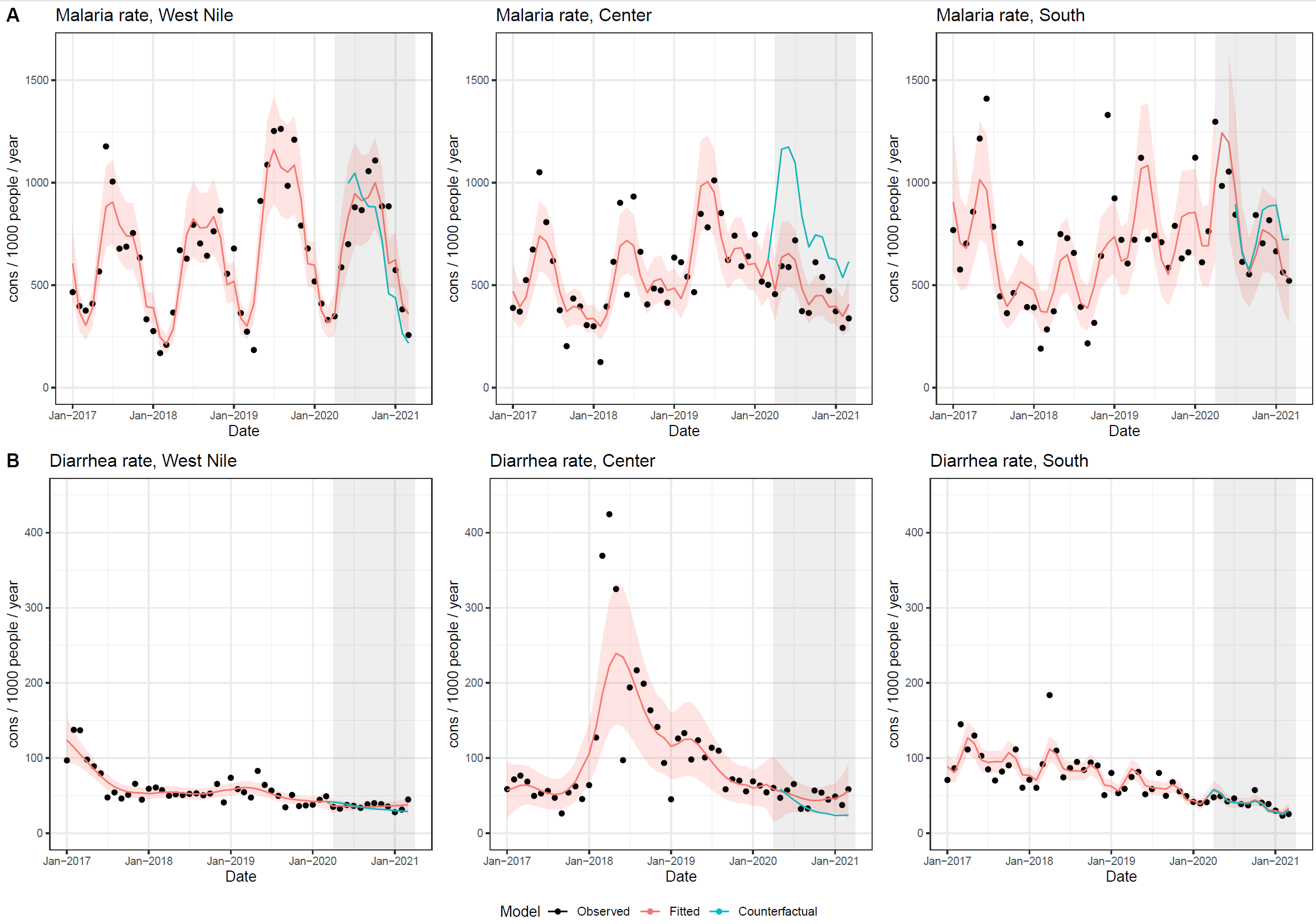


Figure S4: Interrupted time series results for infectious diseases consultations in refugee settlements: malaria (A) and diarrhea (B), by region, Uganda 2017-2021

Figure S5: Interrupted time series results for crude mortality rate in refugee settlements by region, Uganda 2017-2021

## Model fit checks, by variable

### Health utilization rate

|  | Plots | ACF plot | Ljung-Box p-value | Comment |
| --- | --- | --- | --- | --- |
| West Nile | 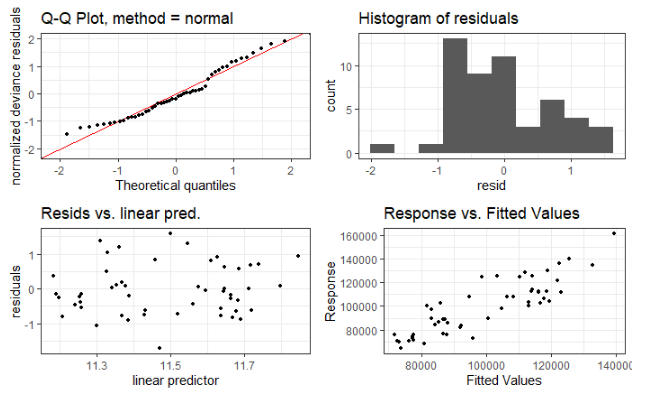 | 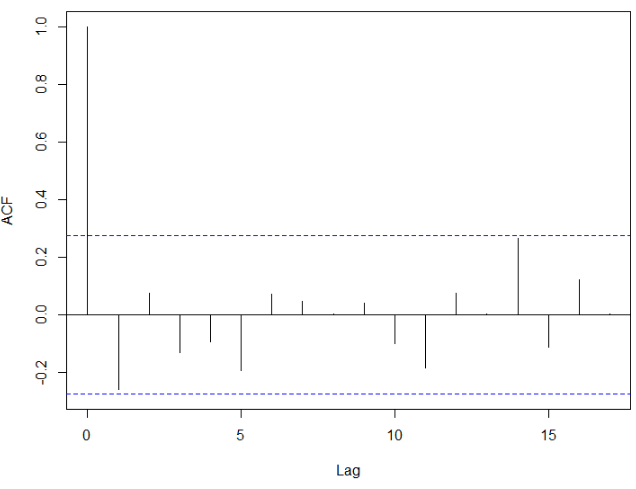 | 0.0580 | Fit seems somewhat reasonable except for the very low quantiles. |
| Center | 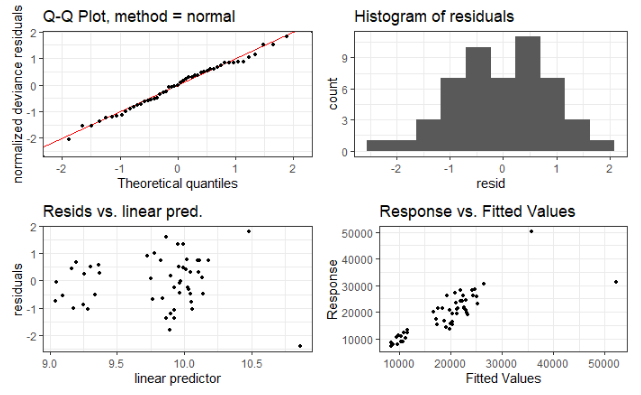 | 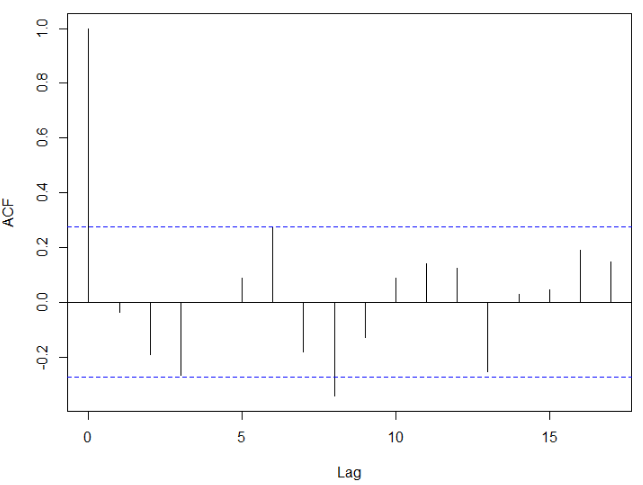  While there is a signal for a lag at 6 months and 8 months, we would expect a shorter lag, if any, for consultations. Hence, we maintain the unlagged analysis as main analysis. | 0.7779 | Fit is good, nothing too concerning, with exception of a few large values that are not captured well by the model. |
| South | 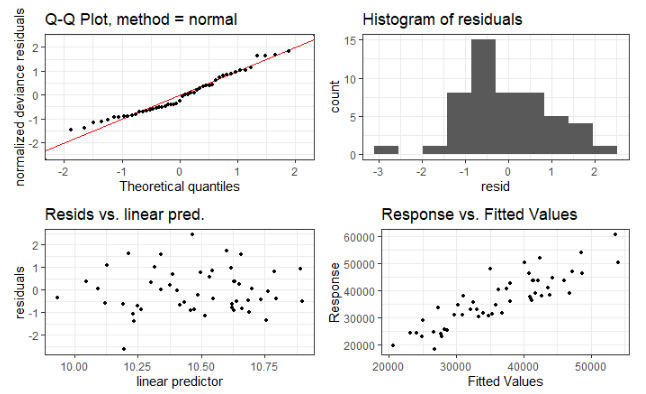 | 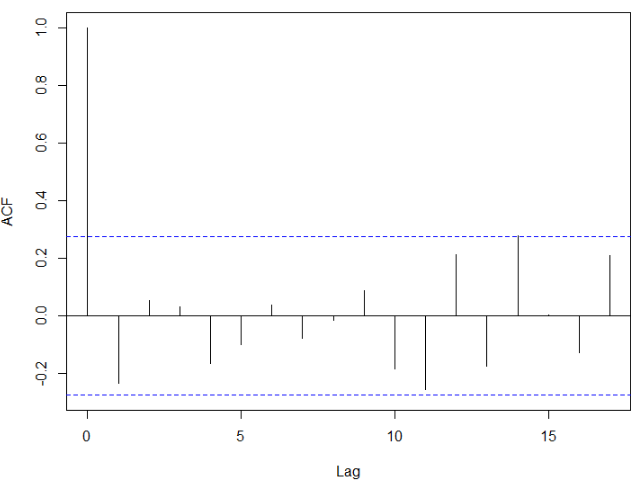 | 0.0882 | Not a bad fit; slight skew in the residuals. |

### ANC1

In Center, there is an extreme value in July 2019 (ANC1 coverage > 5, that is, 500%).


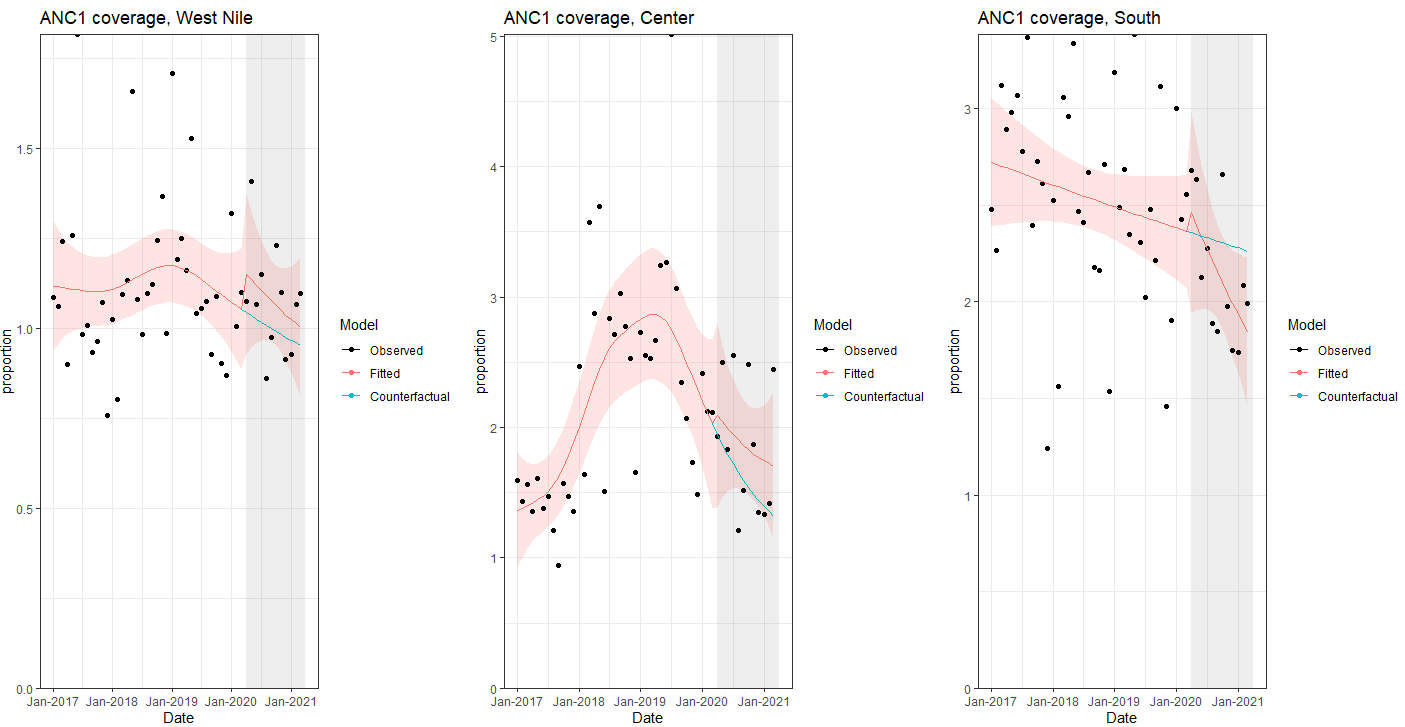
This is because of Kyangwali, where the ANC1 coverage has extreme spikes in January 2018 and July 2019. We remove the two highly influential observations for Center region for the main analysis.


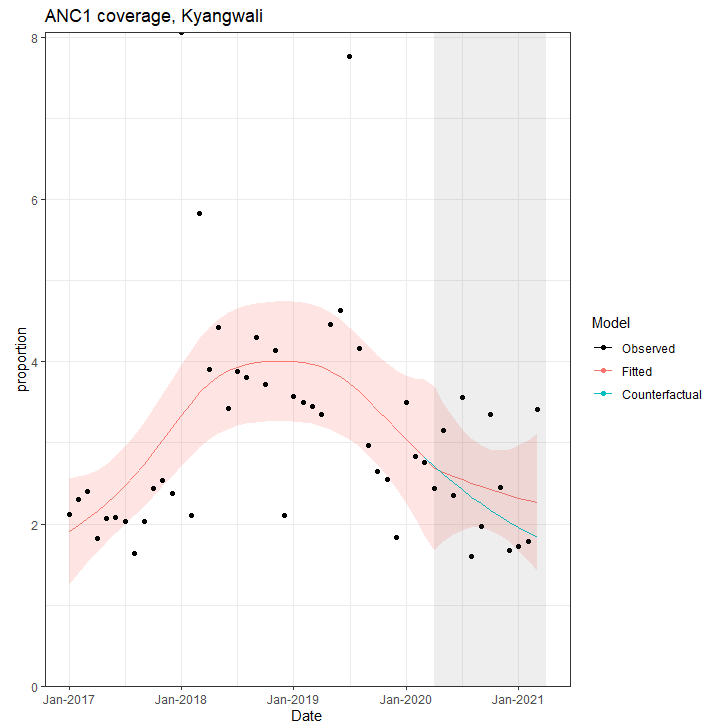


|  | Plots | ACF plot | Ljung-Box p-value | Comment |
| --- | --- | --- | --- | --- |
| West Nile | 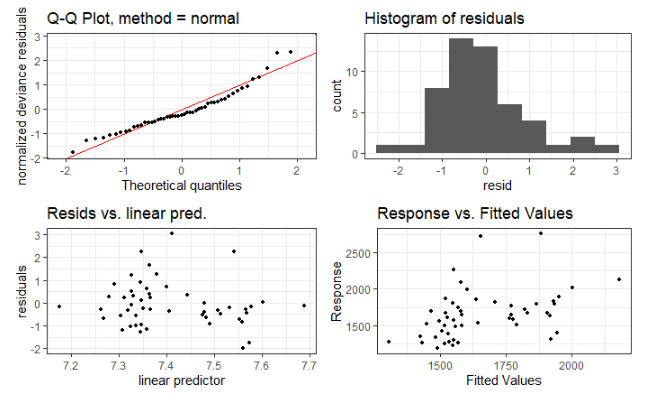 | 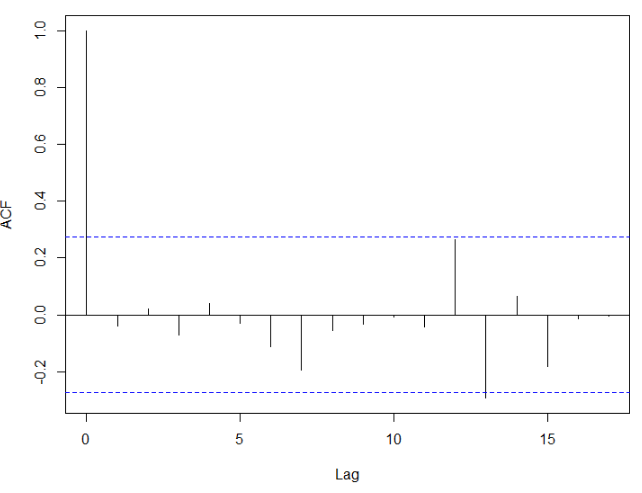 | 0.7697 | No suggestion of significant lag prior to 6 months. Skew in residuals, and QQ plot doesn’t look well in extremes. |
| Center | 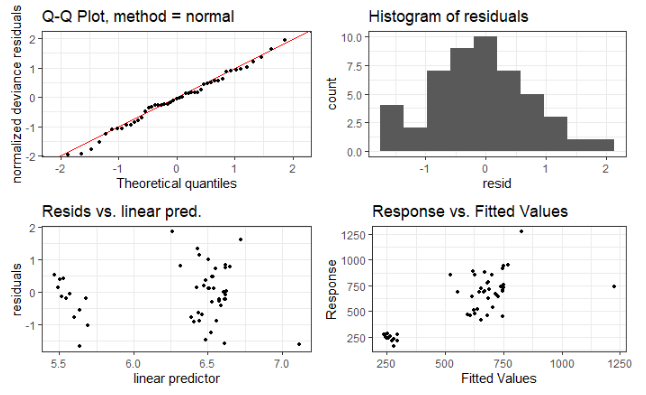 | 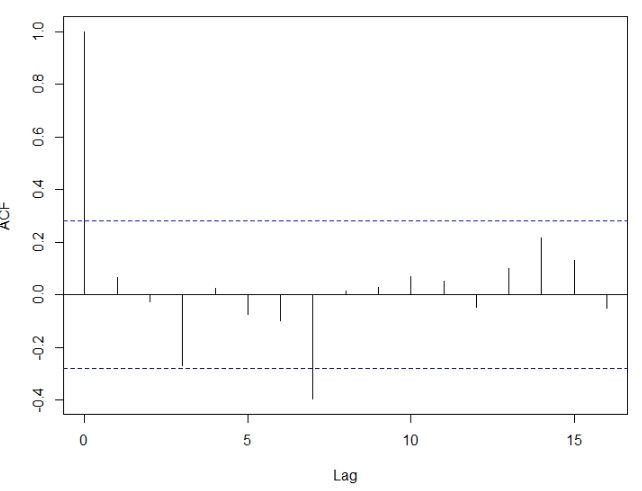 | 0.6342 | Some potential lag at 3 months though ACF is just below cut off; please see below for sensitivity analysis. |
| South | 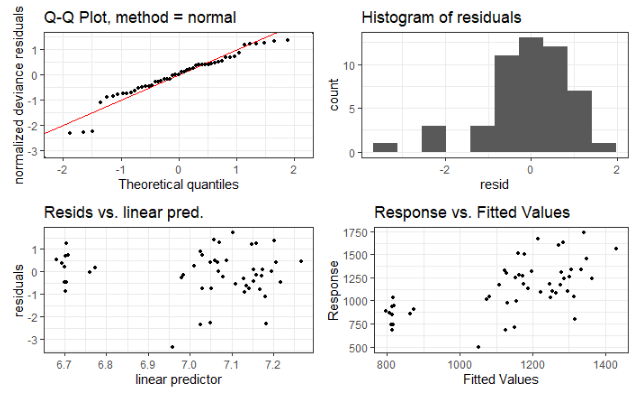 | 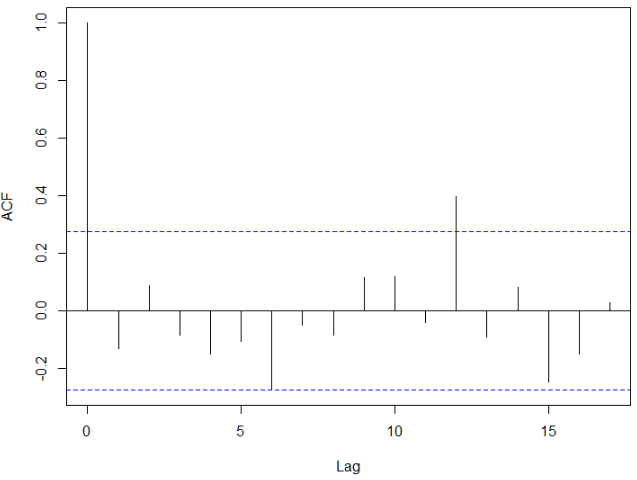 | 0.3408 | Problems in Q-Q plot a the lowest and highest quintiles. No significant lag before 6 months. |

Sensitivity analysis for Center using 3-month lag:

|  | Immediate change | Change in slope |
| --- | --- | --- |
| No lag | 1.07 [0.66 – 1.75] | 1.01 [0.89 – 1.13] |
| 3-month lag | 0.93 [0.57 – 1.52] | 1.00 [0.91 – 1.10] |

The results are not qualitatively different, although there is a slight positive immediate change with no lag, and slight negative immediate change with 3-month lag.

### Skilled deliveries

|  | Plots | ACF plot | Ljung-Box p-value | Comment |
| --- | --- | --- | --- | --- |
| West Nile | 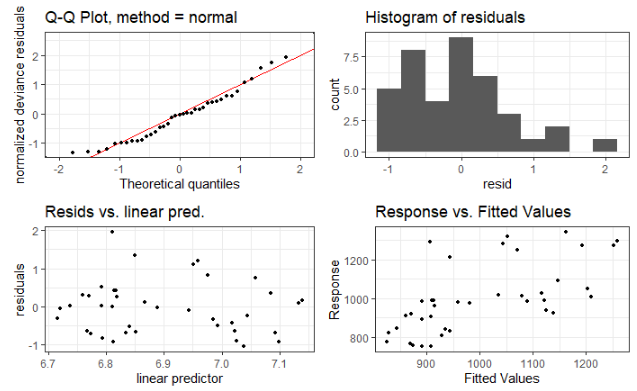 | 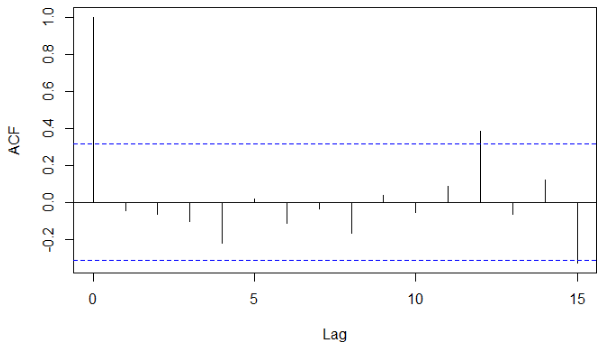 | 0.7804 | Residuals are skewed. There’s not evidence that observations are not independent. |
| Center | 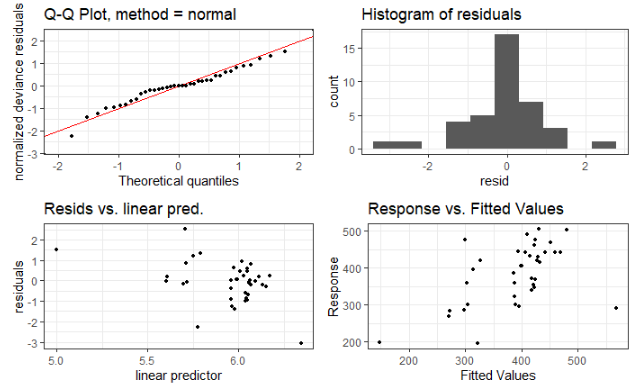 | 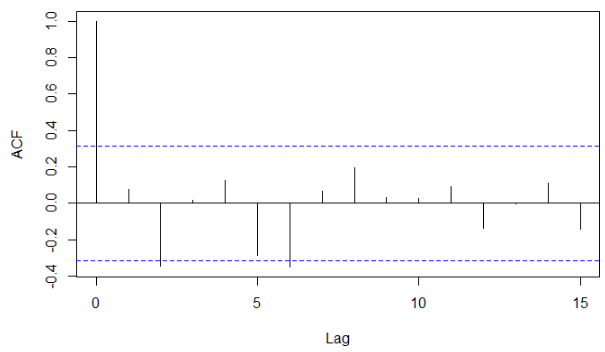 | 0.6228 | Residuals look somewhat normal. There’s no evidence that observations are not independent. There’s a strong lag at 2 months; see below for sensitivity analysis. |
| South | 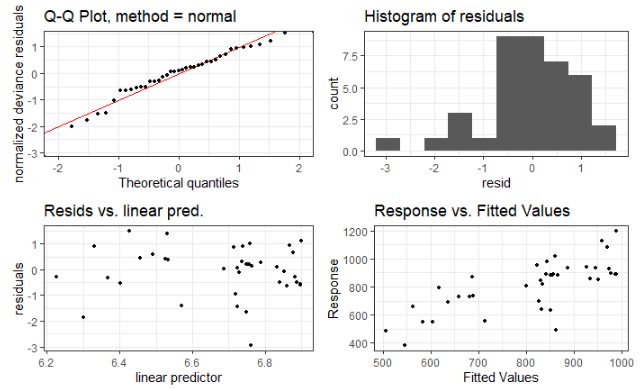 | 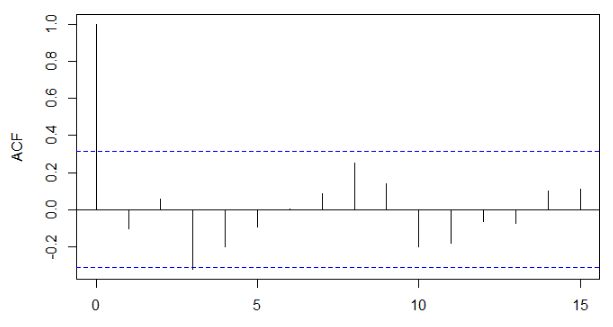 | 0.5065 | Residuals are skewed. There’s evidence of lag at 3 months. See below for sensitivity analysis. |

Sensitivity analysis for Center and South using lag:

|  |  | Immediate change | Change in slope |
| --- | --- | --- | --- |
| Center | No lag | 1.09 [0.81 – 1.46] | 0.99 [0.94 – 1.04] |
|  | 2-month lag | 0.99 [0.94 – 1.02] | 0.98 [0.94 – 1.02] |
| South | No lag | 1.11 [0.83 – 1.49] | 1.00 [0.93 – 1.07] |
|  | 3-month lag | 0.94 [0.69 – 1.28] | 0.99 [0.93 – 1.05] |

We report these lagged results as main analysis.

### Contraceptive prevalence

|  | Plots | ACF plot | Ljung-Box p-value | Comment |
| --- | --- | --- | --- | --- |
| West Nile | 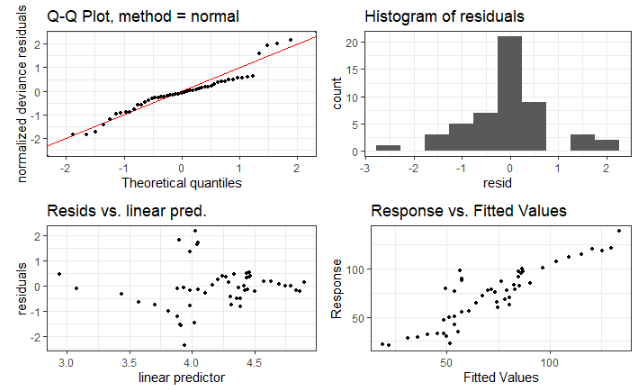 | 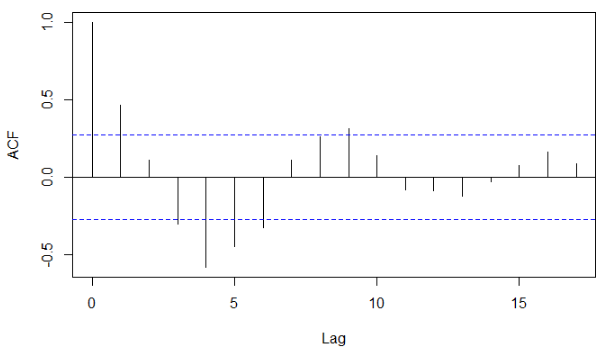 | 0.0006 | Fit is not good. Strongest lag at 4 months; see sensitivity analysis below. |
| Center | 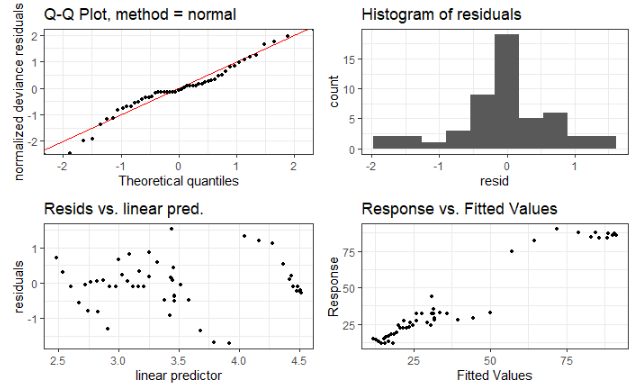 | 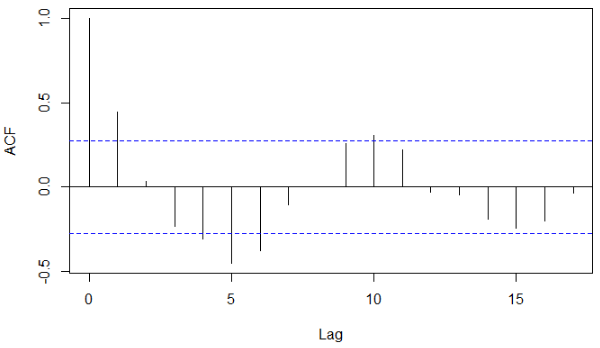 | 0.0010 | Residuals are somewhat normal, but QQ plot is problematic for the lowest quantiles. Strongest lag at 5 months. See sensitivity analysis below. |
| South | 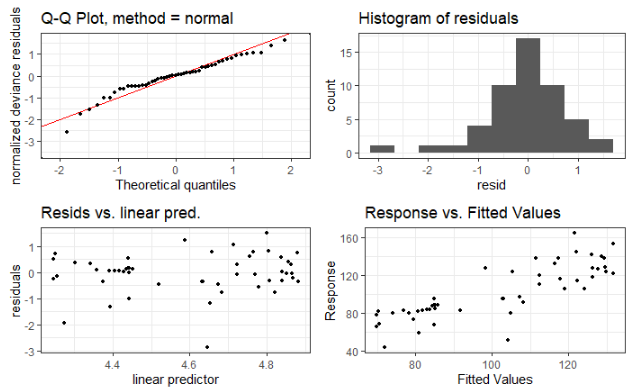 | 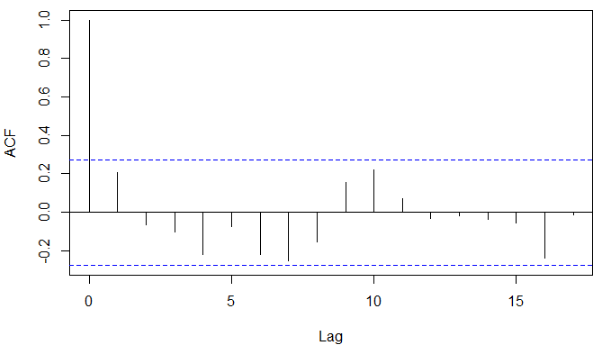 | 0.1285 | Fit is not horrible; residuals are a bit skewed. No indication of lag. |

Sensitivity analysis for West Nile and Center using lag:

|  |  | Immediate change | Change in slope |
| --- | --- | --- | --- |
| West Nile | No lag | 0.865 [0.490 – 1.526] | 1.108 [0.894 – 1.371] |
|  | 4 month lag | 0.929 [0.615 – 1.403] | 1.040 [0.975 – 1.109] |
| Center | No lag | 1.220 [0.652 – 2.283] | 1.100 [0.881 – 1.372] |
|  | 5 month lag | 1.070 [0.545 – 2.100] | 0.980 [0.860 – 1.117] |

We report these lagged results as main analysis.

### DPT1

|  | Plots | ACF plot | Ljung-Box p-value | Comment |
| --- | --- | --- | --- | --- |
| West Nile | 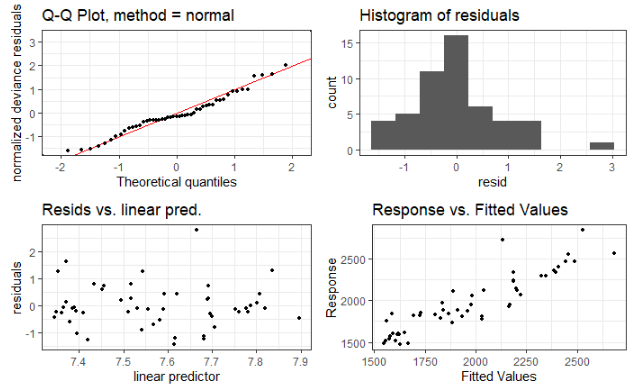 | 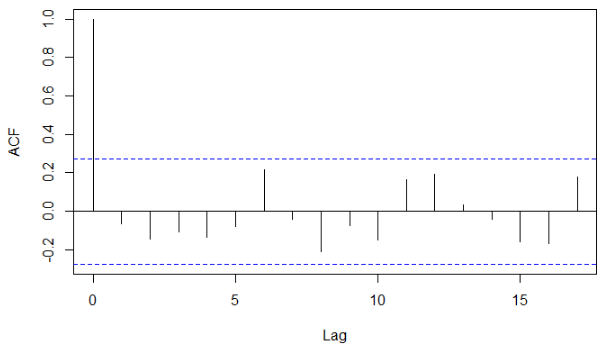 | 0.6360 | Not a bad fit overall; no evidence of lag. |
| Center | 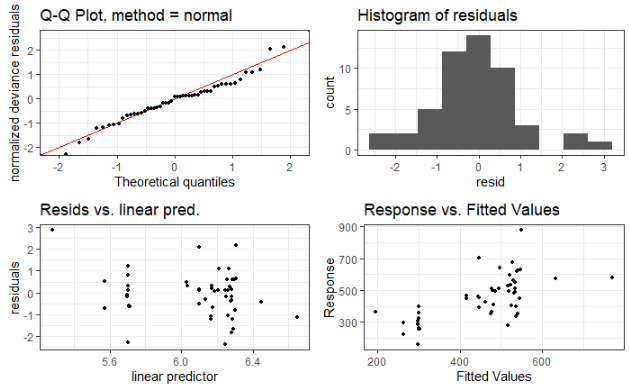 | 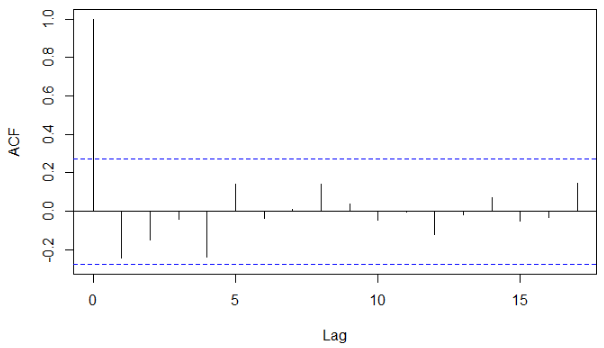 | 0.0762 | Not a bad fit, no evidence of lag |
| South | 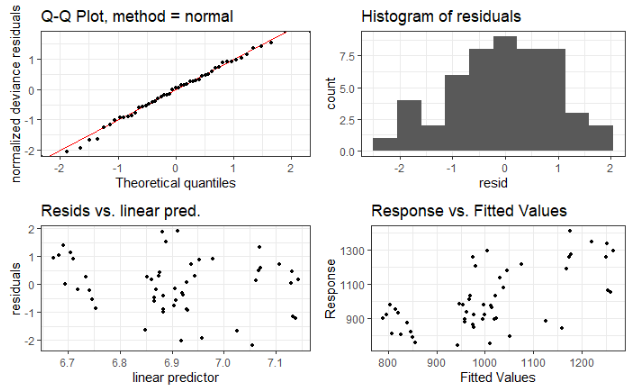 | 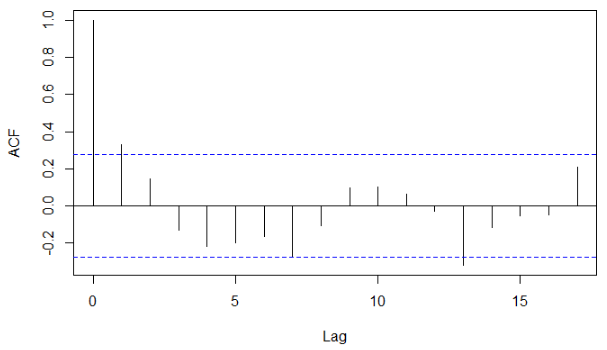 | 0.0152 | Fit is not bad, but evidence of lag at 1 month; see sensitivity analysis below. |

Sensitivity analysis for South using 1 month lag:

|  |  | Immediate change | Change in slope |
| --- | --- | --- | --- |
| South | No lag | 1.079 [0.879 – 1.326] | 1.012 [0.982 – 1.042] |
|  | 1 month lag | 1.092 [0.898 – 1.328] | 1.012 [0.982 – 1.042] |

We report these lagged results as main analysis. There is almost no change.

### URTI

|  | Plots | ACF plot | Ljung-Box p-value | Comment |
| --- | --- | --- | --- | --- |
| West Nile | 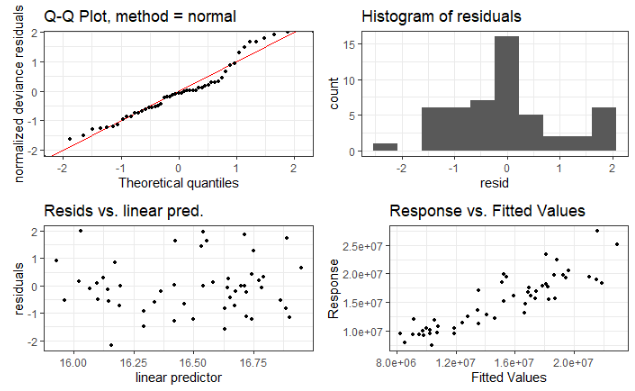 | 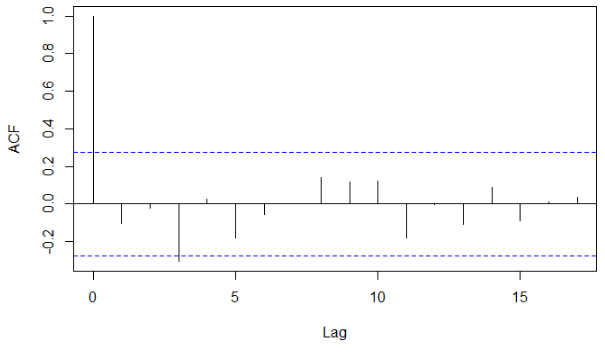 | 0.4509 | Fit is not great, and there’s evidence of 3 month lag. See blow. |
| Center | 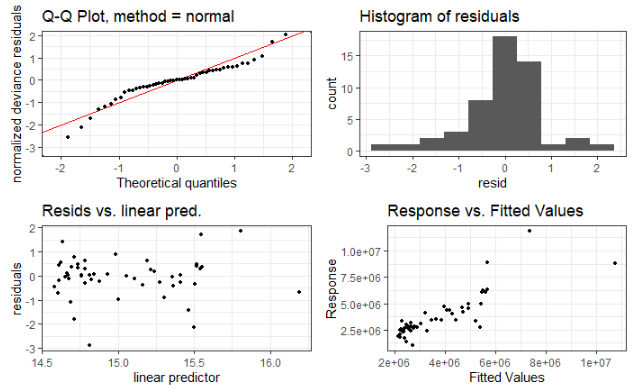 | 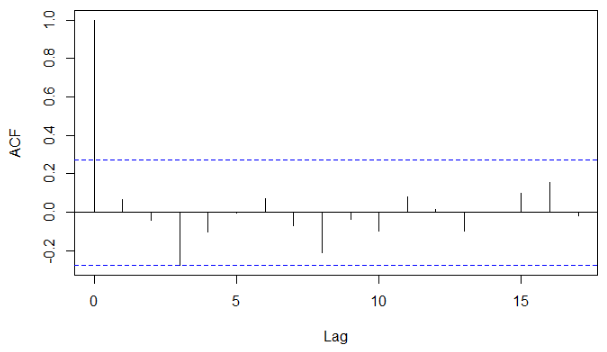 | 0.6220 | Fit is problematic at the edges of quintiles; there’s some evidence of a 3 month lag. See blow. |
| South | 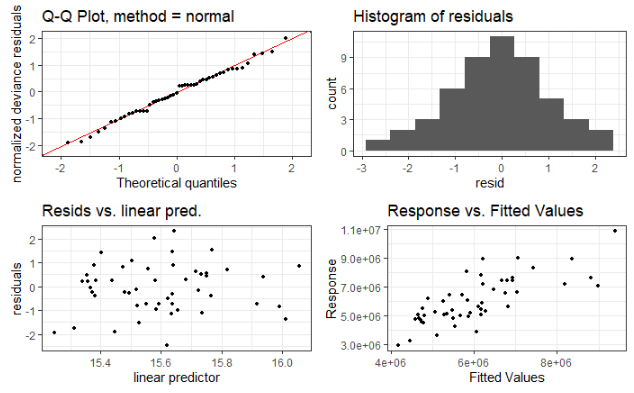 | 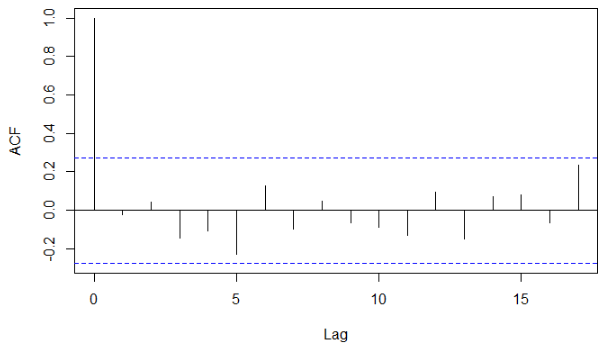 | 0.8618 | Fit is satisfactory; no evidence of lag. Residuals appear normally distributed. |

Sensitivity analysis for West Nile and Center using 3 month lag:

|  |  | Immediate change | Change in slope |
| --- | --- | --- | --- |
| West Nile | No lag | 0.755 [0.575 – 0.992] | 1.036 [0.973 – 1.102] |
|  | 3 month lag | 0.867 [0.650 – 1.157] | 1.059 [0.999 – 1.122] |
| Center | No lag | 0.907 [0.551 – 1.491] | 1.027 [0.900 – 1.172] |
|  | 3 month lag | 0.926 [0.550 – 1.558] | 1.033 [0.927 – 1.151] |

We report these lagged results as main analysis. There is almost no change, although the immediate change result for West Nile is no longer statistically significant.

### LRTI

|  | Plots | ACF plot | Ljung-Box p-value | Comment |
| --- | --- | --- | --- | --- |
| West Nile | 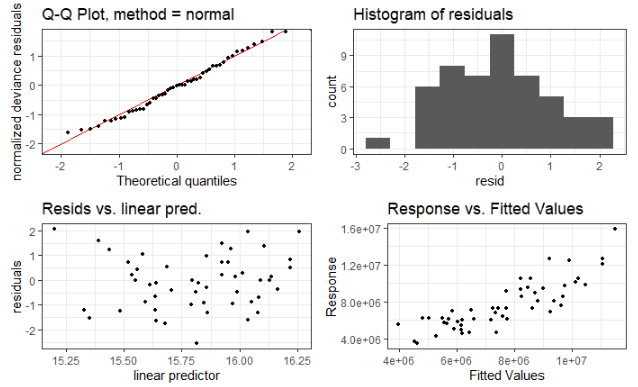 | 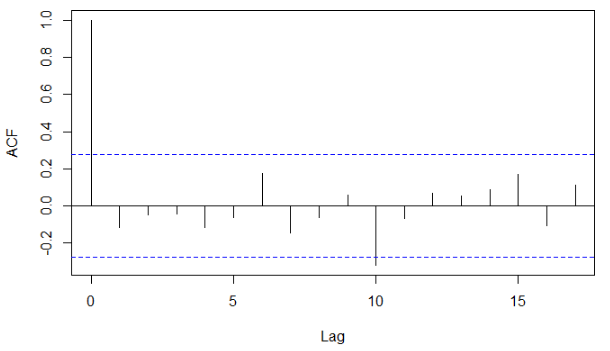 | 0.3901 | Fit is not bad; no strong evidence of lag |
| Center | 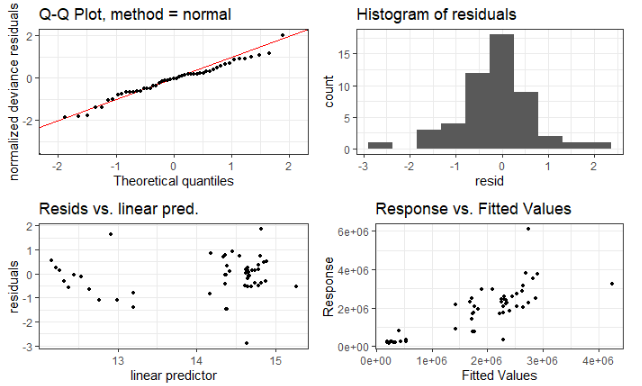 | 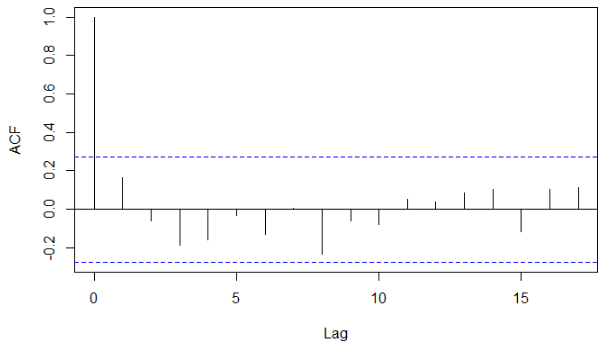 | 0.2327 | Fit is not bad; no strong evidence of lag |
| South | 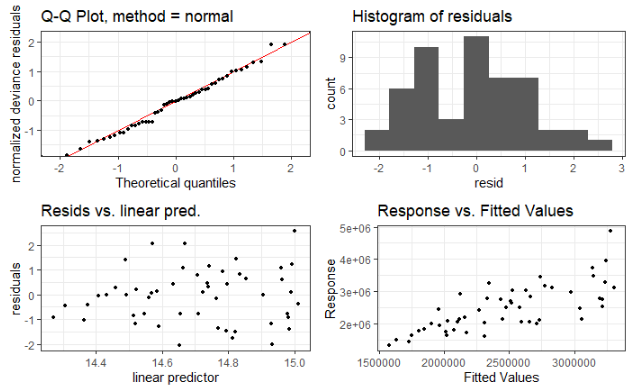 | 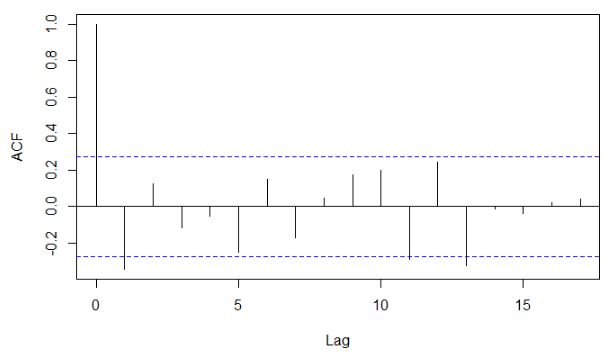 | 0.0116 | Fit is not bad, although there is evidence of lack of independence of residuals, and lag at 1 month. See below. |

Sensitivity analysis for South using 1 month lag:

|  |  | Immediate change | Change in slope |
| --- | --- | --- | --- |
| South | No lag | 0.847 [0.587 – 1.224] | 0.990 [0.877 – 1.117] |
|  | 1 month lag | 0.839 [0.581 – 1.211] | 0.990 [0.877 – 1.117] |

We report these lagged results as main analysis. There is almost no change.

### All RTI

|  | Plots | ACF plot | Ljung-Box p-value | Comment |
| --- | --- | --- | --- | --- |
| West Nile | 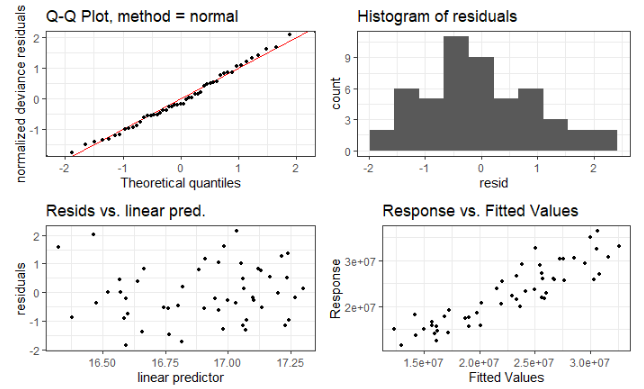 | 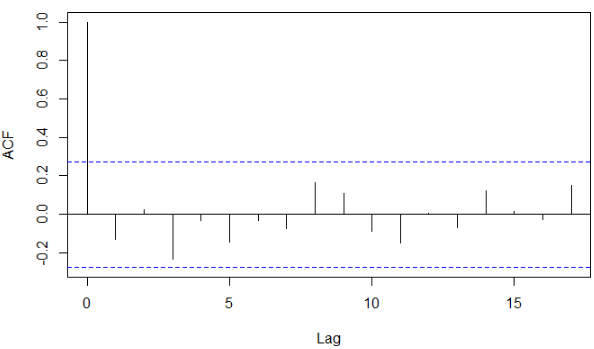 | 0.3313 | Fit is not bad at all, and there’s no strong evidence of lag <6 months |
| Center | 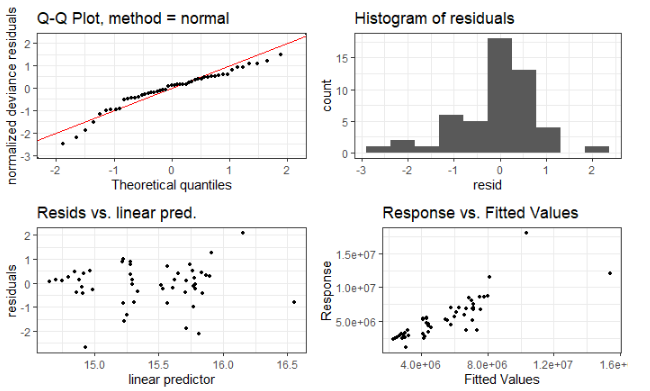 | 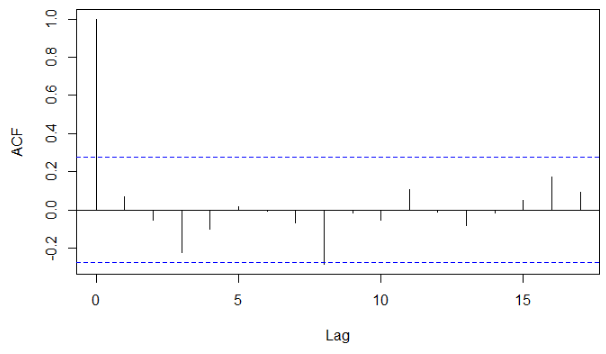 | 0.6074 | Fit is not as good at the edge quintiles; no strong evidence of lag <6 months |
| South | 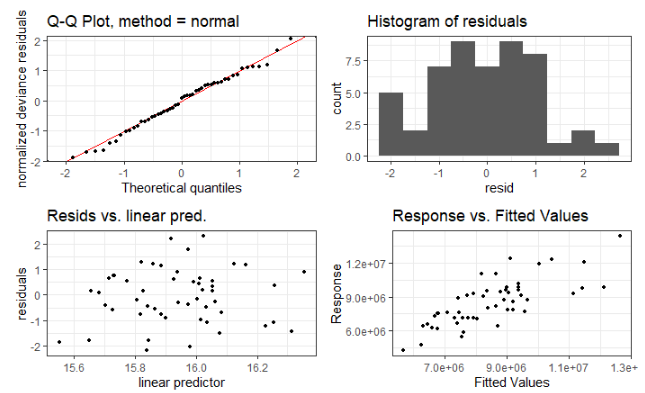 | 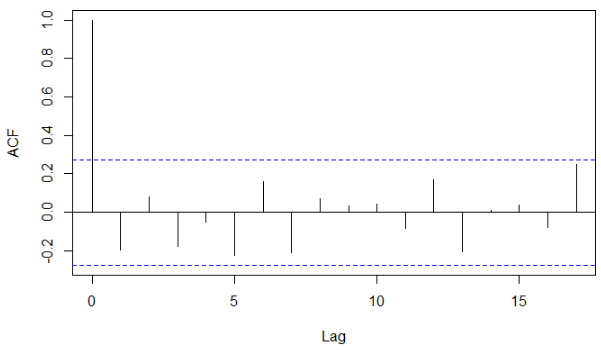 | 0.1516 | Fit is not bad, although residuals don’t look particularly normal. No strong evidence of lag. |

### Malaria

|  | Plots | ACF plot | Ljung-Box p-value | Comment |
| --- | --- | --- | --- | --- |
| West Nile | 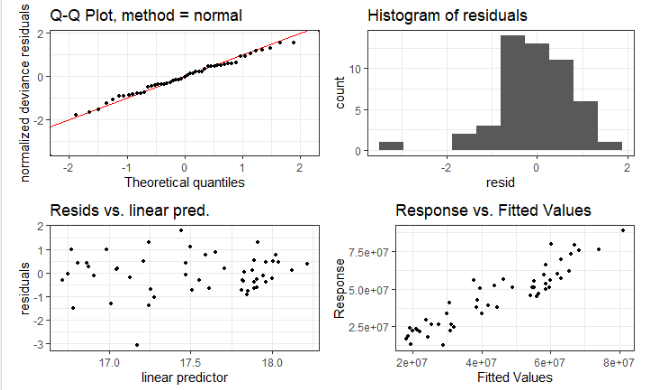 | 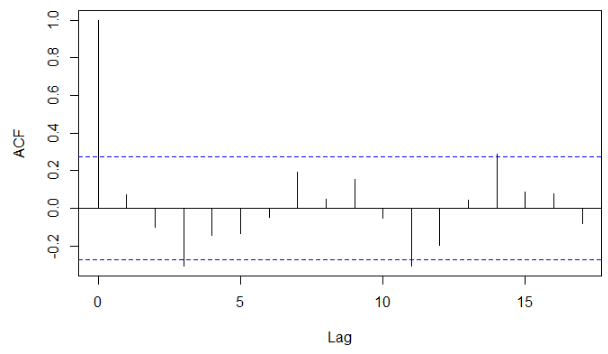 | 0.5992 | Fit is not bad, although there’s a skew in residuals. Suggestion of lag at 3 months (see below). |
| Center | 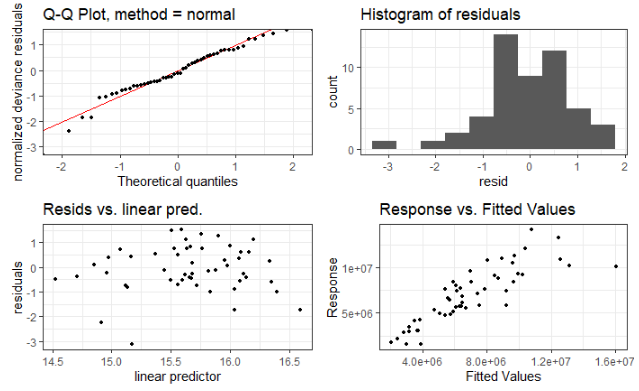 | 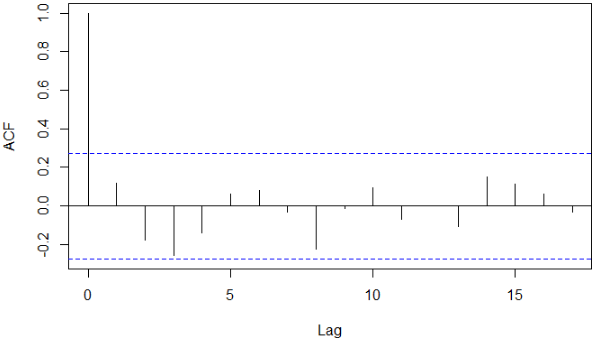 | 0.3854 | Residuals are skewed; no lag suggested, and no evidence that residuals are not independent. |
| South | 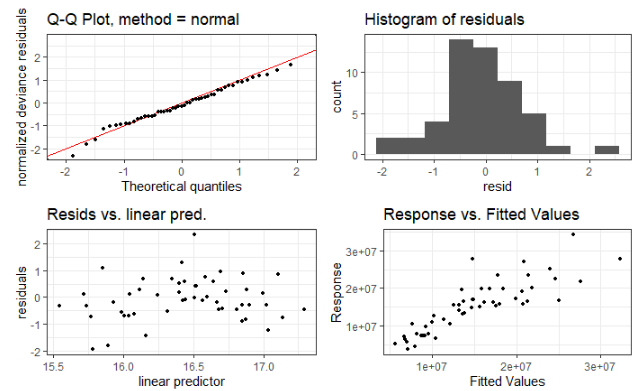 | 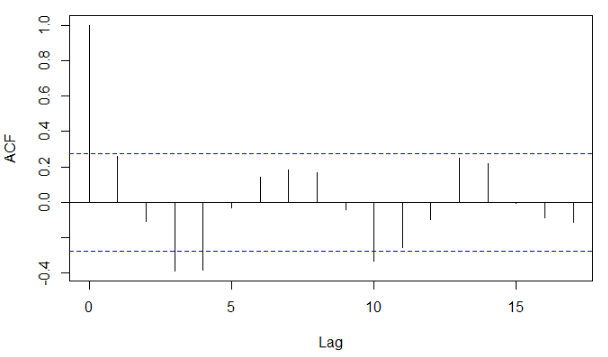 | 0.0552 | Suggest of lag at 4 months (see below). |

Sensitivity analysis for South and West Nile:

|  |  | Immediate change | Change in slope |
| --- | --- | --- | --- |
| West Nile | No lag | 0.669 [0.424 – 1.055] | 1.039 [0.935 – 1.153] |
|  | 3 month lag | 0.838 [0.519 – 1.352[ | 1.079 [0.977 – 1.191] |
| South | No lag | 1.153 [0.604 – 2.202] | 0.947 [0.785 – 1.142] |
|  | 4 month lag | 1.070 [0.551 – 2.080] | 0.955 [0.839 – 1.086] |

We report these lagged results as main analysis. There is almost no change.

### Diarrhea

|  | Plots | ACF plot | Ljung-Box p-value | Comment |
| --- | --- | --- | --- | --- |
| West Nile | 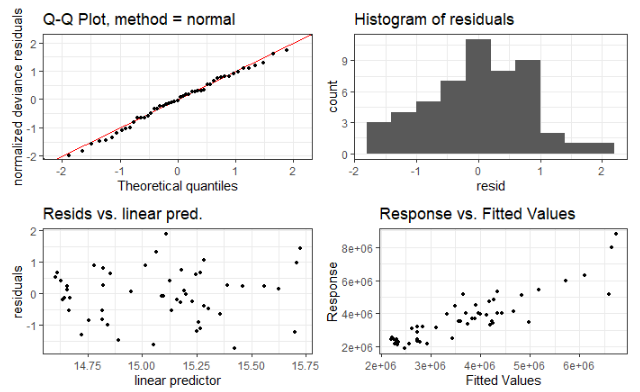 | 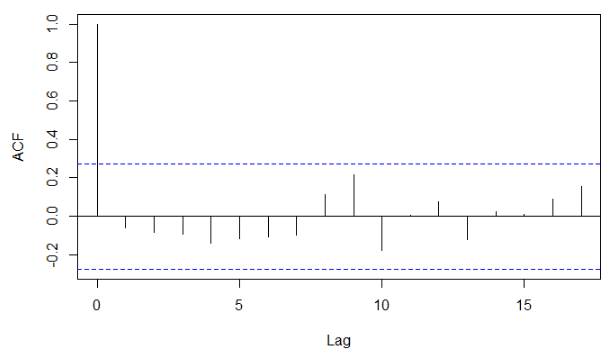 | 0.6532 | The fit is not too bad, and there’s no evidence of lag <6 months; no evidence that residuals are not independent. |
| Center | 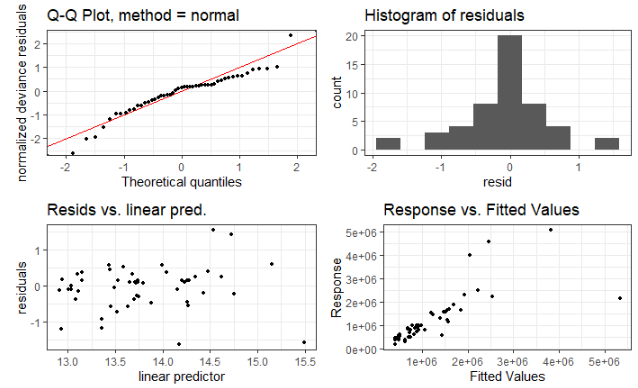 | 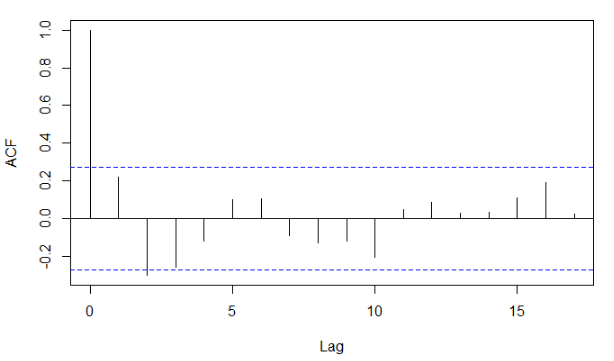 | 0.1091 | The fit for lowest and highest theoretical quantiles is not good; evidence of lag at 2 months (see below). We observe some high observations in 2018 that seem unusually high. |
| South | 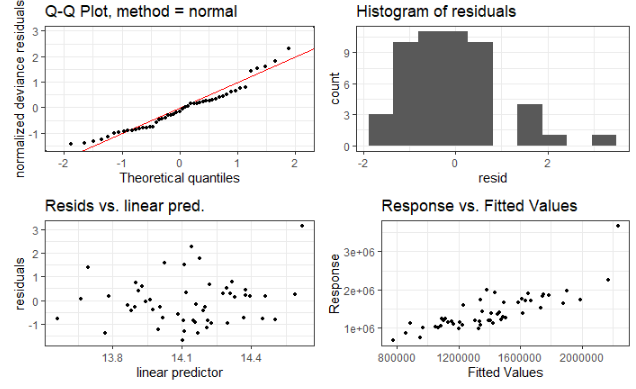 | 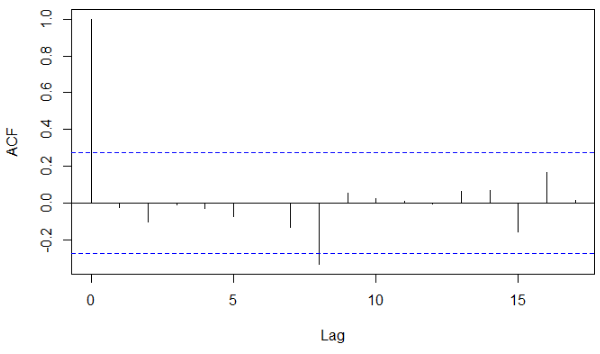 | 0.8667 | No evidence of lag <6 months. Fit is not good at the edge quintiles. There is an unusually high month of observations in 2018. |

Sensitivity analysis for Center:

|  |  | Immediate change | Change in slope |
| --- | --- | --- | --- |
| Center | No lag | 0.845 [0.288 – 2.480] | 1.101 [0.737 – 1.643] |
|  | 2 month lag | 0.951 [0.325 – 2.784] | 1.093 [0.779 – 1.534] |

We report these lagged results as main analysis.

### Mortality

|  | Plots | ACF plot | Ljung-Box p-value | Comment |
| --- | --- | --- | --- | --- |
| West Nile | 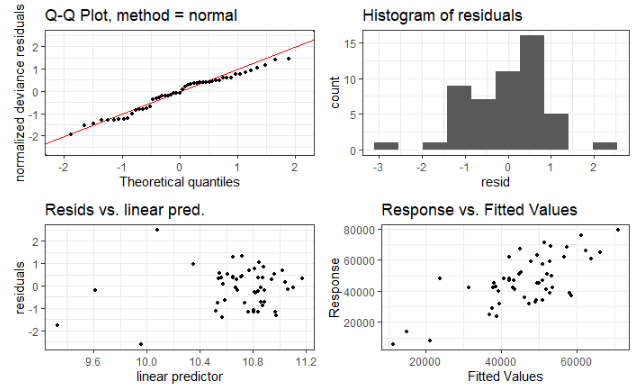 | 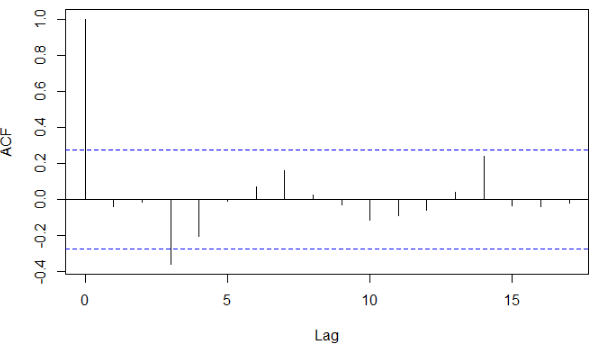 | 0.7735 | Fit is not bad, but some evidence of non-normal distribution of residuals, and presumed lag at 3 months (see below). |
| Center | 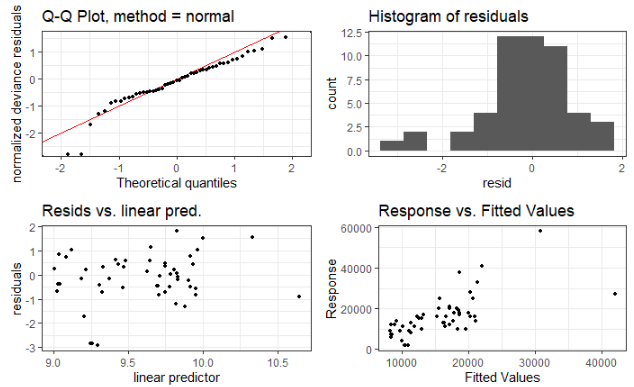 | 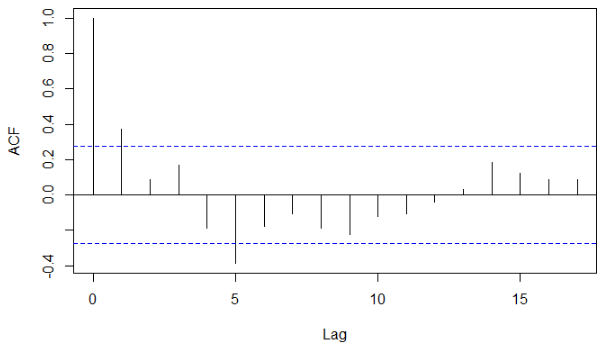 | 0.0063 | Some evidence of non-normality of residuals, poor fit of Q-Q plot at the lowest quintiles. Suggested lag at 5 months (see below). |
| South | 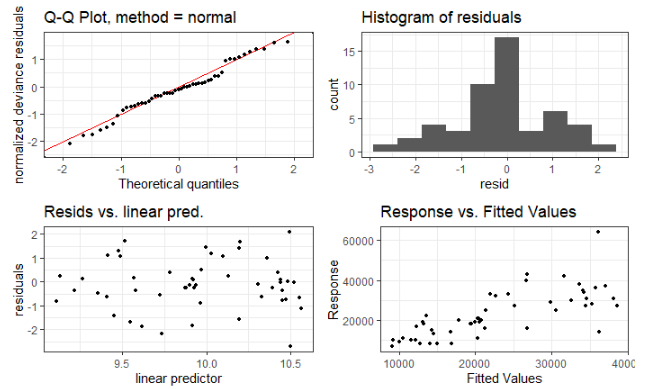 | 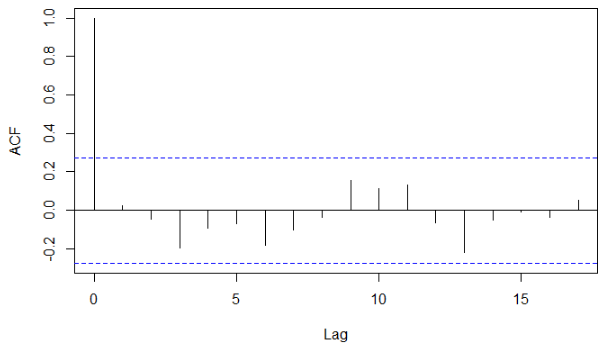 | 0.8530 | Somewhat normal residuals, relatively good fit. No strong suggestion of lag. |

Sensitivity analysis for West Nile and Center:

|  |  | Immediate change | Change in slope |
| --- | --- | --- | --- |
| West Nile | No lag | 1.340 [0.686 – 2.618] | 0.947 [0.769 – 1.167] |
|  | 3 month lag | 1.649 [0.881 – 3.088] | 0.891 [0.781 – 1.016] |
| Center | No lag | 0.966 [0.462 – 2.021] | 0.952 [0.847 – 1.072] |
|  | 5 month lag | 0.746 [0.324 – 1.717] | 0.989 [0.875 – 1.119] |

The results are not qualitatively different. We report the lagged ones in main analysis
